# *In vitro* infection models to study fungal–host interactions

**DOI:** 10.1093/femsre/fuab005

**Published:** 2021-02-01

**Authors:** Antonia Last, Michelle Maurer, Alexander S. Mosig, Mark S. Gresnigt, Bernhard Hube

**Affiliations:** Department of Microbial Pathogenicity Mechanisms, Leibniz Institute for Natural Product Research and Infection Biology—Hans Knoell Institute, Beutenbergstrasse 11a, 07745, Jena, Germany; Center for Sepsis Control and Care (CSCC), Jena University Hospital, Am Klinikum 1, 07747, Jena, Germany; Institute of Biochemistry II, Jena University Hospital, Nonnenplan 2,07743, Jena, Germany; Center for Sepsis Control and Care (CSCC), Jena University Hospital, Am Klinikum 1, 07747, Jena, Germany; Institute of Biochemistry II, Jena University Hospital, Nonnenplan 2,07743, Jena, Germany; Junior Research Group Adaptive Pathogenicity Strategies, Leibniz Institute for Natural Product Research and Infection Biology—Hans Knoell Institute, Beutenbergstrasse 11a, 07745, Jena, Germany; Department of Microbial Pathogenicity Mechanisms, Leibniz Institute for Natural Product Research and Infection Biology—Hans Knoell Institute, Beutenbergstrasse 11a, 07745, Jena, Germany; Institute of Microbiology, Friedrich Schiller University, Neugasse 24, 07743, Jena, Germany

**Keywords:** *in vitro* model, fungal–host interaction, *Aspergillus*, *Candida*, *Histoplasma*, *Cryptococcus*

## Abstract

Fungal infections (mycoses) affect over a billion people per year. Approximately, two million of these infections are life-threatening, especially for patients with a compromised immune system. Fungi of the genera *Aspergillus*, *Candida*, *Histoplasma* and *Cryptococcus* are opportunistic pathogens that contribute to a substantial number of mycoses. To optimize the diagnosis and treatment of mycoses, we need to understand the complex fungal–host interplay during pathogenesis, the fungal attributes causing virulence and how the host resists infection via immunological defenses. *In vitro* models can be used to mimic fungal infections of various tissues and organs and the corresponding immune responses at near-physiological conditions. Furthermore, models can include fungal interactions with the host–microbiota to mimic the *in vivo* situation on skin and mucosal surfaces. This article reviews currently used *in vitro* models of fungal infections ranging from cell monolayers to microfluidic 3D organ-on-chip (OOC) platforms. We also discuss how OOC models can expand the toolbox for investigating interactions of fungi and their human hosts in the future.

## Teaser

From basic to complex: *in vitro* models to study interactions between human fungal pathogens and their host.

## INTRODUCTION

Human fungal infections lead to approximately 1.5 million deaths worldwide each year, but receive little attention compared with malaria or tuberculosis, which kill a similar number of people on an annual basis (Brown *et al*. 2012; Bongomin *et al*. 2017). Over 70% of deaths resulting from fungal infections can be attributed to fungi of the genera *Aspergillus*, *Candida*, *Cryptococcus* and *Histoplasma* (Brown *et al*. 2012). These opportunistic fungal pathogens are either normal commensals of the human microbiota or reside in the environment, resulting in constant exposure to pathogenic fungi for humans. Even in immunocompetent human hosts, superficial fungal infections are widespread. Among them, fungal skin diseases are the most common health complications (Vos *et al*. 2012), and vulvovaginal candidiasis (VVC) affects approximately 70% of women (Gonçalves *et al*. 2016; Rosati *et al*. 2020). Such infections are often connected to an imbalance of the bacterial microbiota, for example, after the use of antibiotics that favor fungal overgrowth (Weiss and Hennet 2017)). In addition to superficial infections, opportunistic fungal pathogens can also cause severe life-threatening systemic infections under certain predispositions, like surgery, stem cell transplantation, chemotherapy or HIV/AIDS (Perlroth, Choi and Spellberg 2007; Polvi *et al*. 2015; Vallabhaneni and Chiller 2016). Considering their clinical significance, suitable models to study opportunistic fungal infections are essential for obtaining insights into disease pathogenesis. Ideally, these models allow the dissection of the molecular details of host–pathogen interactions under physiologically relevant conditions. They should provide sufficient complexity to mimic the different types and stages of infections and predispositions of the host. These models should also be suitable to test experimental therapeutic interventions and allow the evaluation of clinically relevant biomarkers. Here, we review currently used *in vitro* models to study molecular mechanisms of fungal infections caused by common fungal pathogens, including *Aspergillus fumigatus*, *Candida* spp., *Cryptococcus neoformans* and *Histoplasma capsulatum*, and provide an outlook about models that will likely expand our toolbox to study fungal–host interactions in the near future.

## DISEASE MODELING

**Figure 1.**
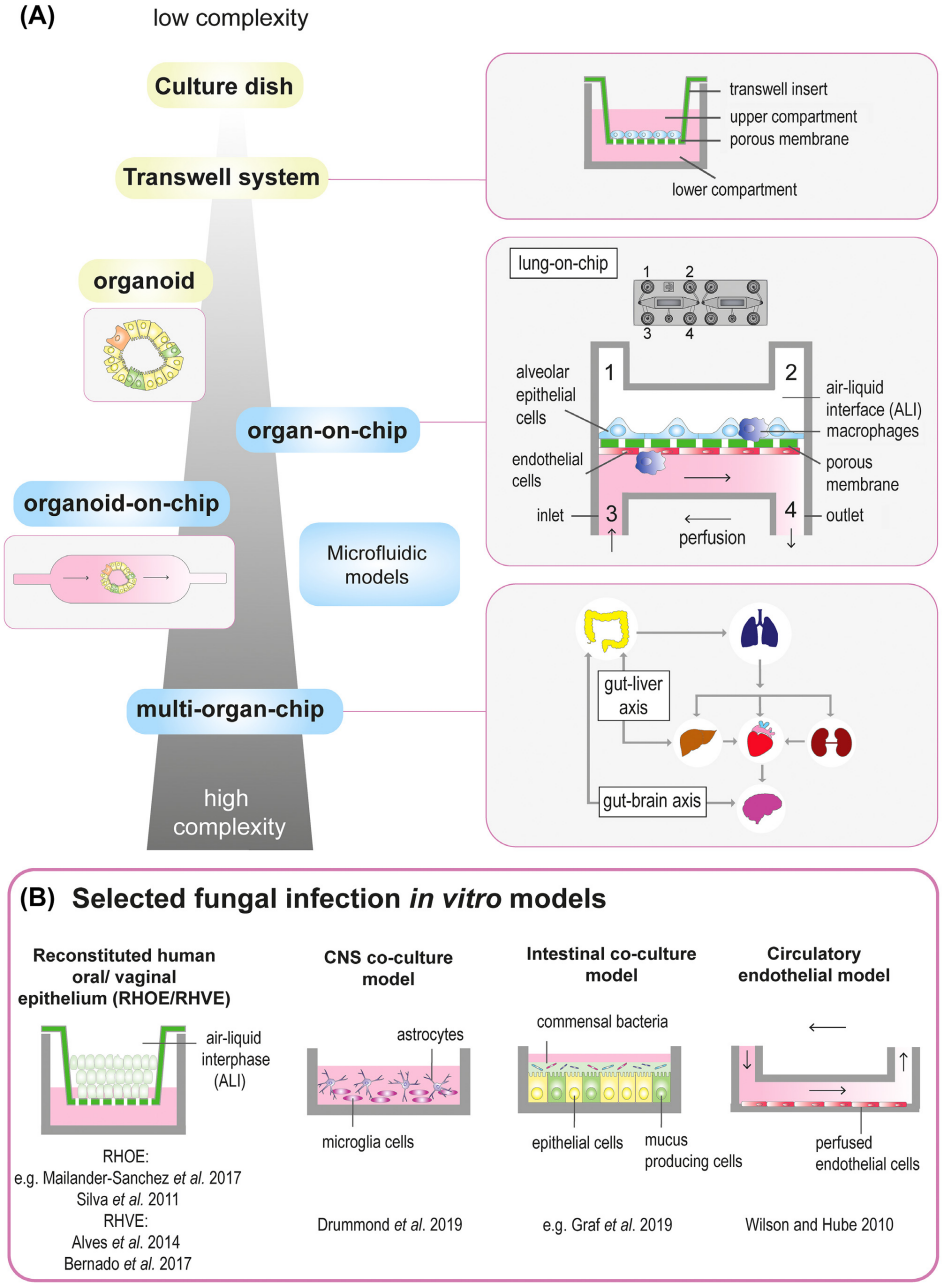
**(A)**Evolution of *in vitro* models from low to high complexity. Culture dish: one cell type cultured in media. Transwell system: transwell inserts separate the culture area into an upper and lower compartment; cells are cultured under static conditions on a porous membrane allowing apical-basal polarization. Organoid: 3D miniature organ generated out of intestinal stem cells. Organ-on-chip (example): 3D lung on-chip model on a microfluidic biochip holding a porous membrane and two individually accessible channels with one inlet and outlet each; pulmonary epithelial cells are cultured in the upper compartment in an air–liquid interface; and endothelial cells in the lower compartment are perfused with cell culture medium enabling the removal of metabolites. Organoid-on-chip: maturation of organoids within a dynamic culture environment. Principle of a multi-organ-on-chip: interconnected organ-on-chip models of gut and liver, or gut and brain or other combinations of lung, intestine, liver, brain and/or kidneys. Such combinations can, for example, mimic certain steps of fungal dissemination throughout the body. The intestine and lung serve as primary infection sites. **(B)**Selected *in vitro* models to study host–fungal interactions. 3D reconstituted human oral (RHOE) or vaginal (RHVE) epithelium grown at an air–liquid interface. Central nervous system (CNS) co-culture model including microglia cells and astrocytes. Intestinal co-culture model including epithelial cells, goblet cells and bacteria. Circulatory model with perfused endothelial cells.

To study fungal pathogens and their related diseases, a wide range of models can be used. Commonly, host–pathogen interactions are investigated in animal model organisms such as mice, rats, fish, insects or worms. *In vivo* models offer the advantage to study host–pathogen interactions in a whole organism, providing the most complex interactions that can be achieved experimentally. However, in addition to critical ethical issues associated with the use of animal models (Robinson *et al*. 2019), the translation of results from animal experiments to human disease can be hampered by differences in physiology. Another approach is the use of tissue samples or organs from living organisms and their culture in an *ex vivo* environment that resembles *in vivo* conditions. These *ex vivo* models offer the advantage that conditions can be easily manipulated and are often easier to handle than living organisms. A broad overview of *ex vivo* models to study fungal infection is given by Maciel Quatrin *et al*. (2019). *In vitro* experiments are also performed outside of the natural biological environment. Primary cells isolated from tissues and biopsies can be cultured for a limited time or can be immortalized and cultured as cell lines. *In vitro* models may lack the complexity of *in vivo* models, but allow ample control over external growth conditions of cells concerning O_2_ and CO_2_ saturation, temperature, pH and nutrients. Moreover, it is relatively easy to manipulate as well as to quantitatively and qualitatively assess the metabolism, transcription and protein function of cells, making it possible to work in and test conditions that cannot be studied in *in vivo* models. It is also possible to introduce or omit different cell types to study the individual impact of different kinds of cells within the system. *In vitro* models (Fig. 1) range from monolayers in well plates, to transwell systems, 3D tissue structures and complex organ-on-chip (OOC) models (Mosig 2017), which are used to mimic several organs such as the liver (Groger *et al*. 2016; Jang *et al*. 2019), lung (Benam *et al*. 2016; Deinhardt-Emmer *et al*. 2020) and gut (Shin and Kim 2018; Maurer *et al*. 2019). OOC models represent the smallest functional entity of an organ as well as a versatile and promising resource to study host–pathogen interactions (Ahadian *et al*. 2018). However, each model has its specific advantages and disadvantages. The most suitable model is the one that meets the actual needs with high predictability and robustness, depending on the pathogen, the host and the questions to be answered.

**Figure 2.**
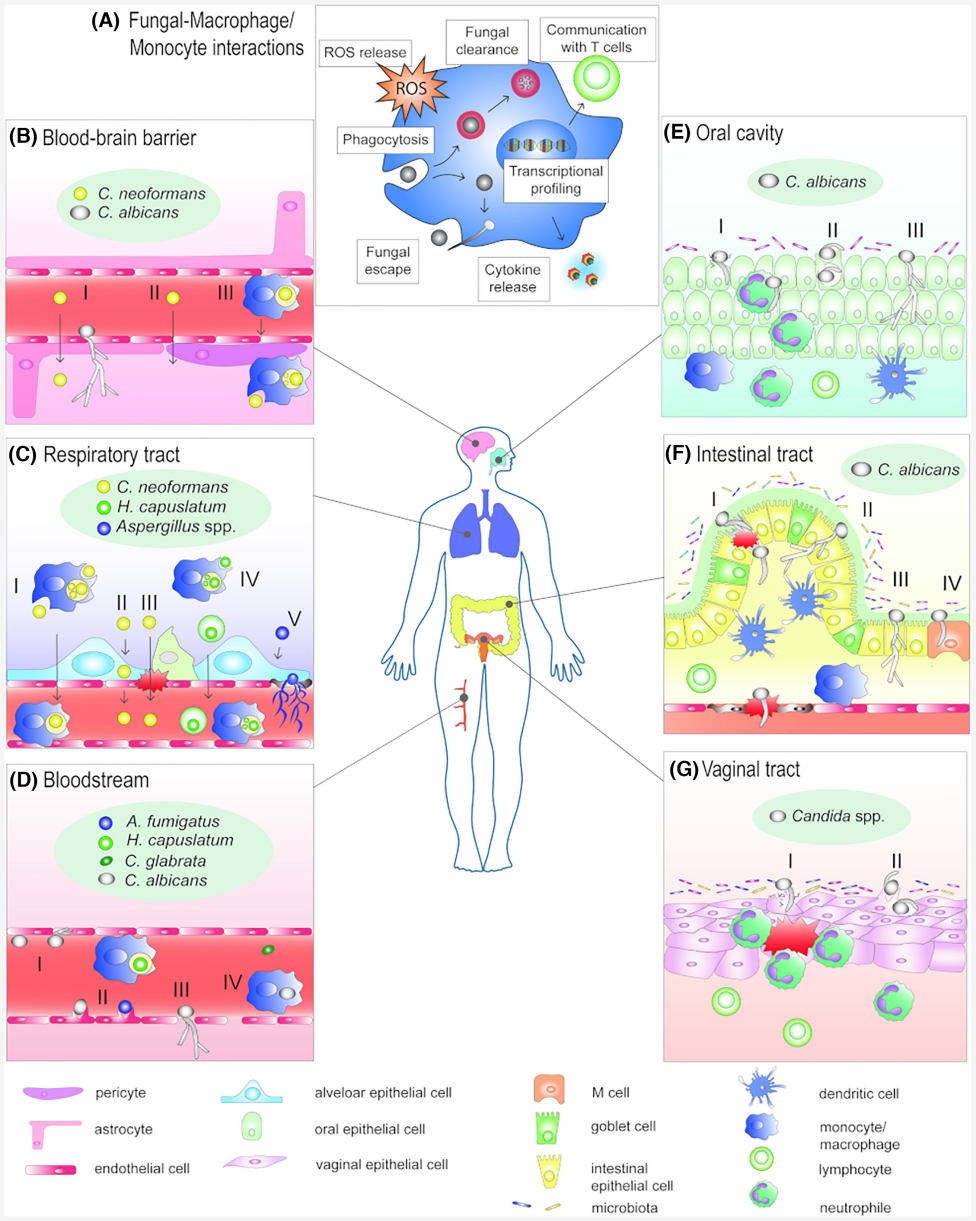
Fungal–host interactions during fungal diseases that are mimicked by *in vitro* infection models discussed in this review. **(A)** Fungal–monocyte/macrophage interactions resulting in several effector mechanisms that contribute to immunity against fungal infections (ROS: reactive oxygen species). **(B)***C. neoformans* and *C. albicans* can cross the BBB via transcytosis **(I);***C. neoformans* can overcome the barrier paracellularly **(II)** or use macrophages as shuttles (macrophages as ‘Trojan horse’) **(III)**. **(C)** In the lung, *C. neoformans* and *H. capsulatum* induce their own phagocytosis by innate immune cells; they can replicate intracellularly and use host cells as shuttles to reach the blood stream and subsequently escape **(I** and **IV)**; evasion of *C. neoformans* via transcytosis **(II)** or crossing of *C. neoformans* through a compromised epithelium **(III)**. *Aspergillus* spp. form hyphae, can invade endothelial cells and enter the bloodstream **(V)**. **(D)***Candida* spp. can escape the blood circulation after adhesion to endothelial cells **(I)**. *Candida* spp. and *A. fumigatus* can be endocytosed **(II)**; *Candida* spp. can also use fenestrated endothelium as an escape route **(III)** or use leukocytes as shuttles **(IV)**. **(E)** In the oral cavity, *C. albicans* hyphae can actively penetrate the epithelium **(I)** and/or invade via induced endocytosis **(II)** or translocate paracellularly **(III). (F)** In the intestine, *C. albicans* can actively penetrate the epithelium by hyphal growth **(I)**, translocate paracellularly **(II)**, invade without damaging the host cell **(III)** or translocate via M cells by inducing endocytosis **(IV)**. **(G)** In the vaginal tract, *C. albicans* hyphae can actively penetrate the epithelium **(I)** or invade via induced endocytosis **(II)**, thereby attracting neutrophils.

We discuss the fungal–host interactions in different biological niches (Fig. 2). We review *in vitro* models used to mimic infection routes and highlight relevant findings that contributed to expand our knowledge on fungal infections. Because the immune system plays a major role during fungal infections, the interplay of fungi and immune cells is discussed in the first part, followed by sections covering the respiratory tract, the gastrointestinal tract, the vaginal mucosa, the bloodstream and the blood–brain barrier (BBB).

## STUDYING FUNGAL INTERACTIONS WITH THE IMMUNE SYSTEM

A properly functioning immune system is crucial for resistance against infections with fungal pathogens. Individuals with a compromised immune system are more susceptible to invasive fungal diseases, whereas detrimental, improper or hypersensitive immune reactions can also contribute to disease (Romani 2004; Wheeler, Limon and Underhill 2017). Thus, a protective host response against opportunistic fungal pathogens has to be specific, tightly regulated and effective. However, pathogenic fungi have evolved a series of mechanisms to deal with and evade the immune system. Knowledge of both aspects is crucial for the design of therapeutic strategies aiming to strengthen appropriate responses and suppress detrimental ones (Armstrong-James *et al*. 2017). We will discuss (i) the different immune cells involved in antifungal host defense, (ii) the different roles these cells play in antifungal immunity and (iii) different models and readouts that can be used to study the efficiency of the host response to pathogenic fungi.

### Immune cells involved in antifungal host defense

A healthy and efficient immune system is fundamental to cope with the environmental fungi we encounter on a daily basis and to deal with the fungi we harbor as commensals. This antifungal immunity relies on the innate immune system represented by cells such as macrophages, monocytes, neutrophils, natural killer (NK) cells and dendritic cells (DCs) as well as the adaptive immune system, in particular on T helper cell responses. The importance of these different types of immune cells becomes apparent when they are dysfunctional or absent. For example, a compromised innate immune system due to immunosuppressive therapy predisposes not only to invasive candidiasis (Lionakis 2014) but also aspergillosis (Herbrecht *et al*. 2012). While the innate immune system plays a role in host defense against cryptococcosis (Voelz and May 2010), patients with a compromised adaptive immune response due to HIV infections are particularly susceptible (Warkentien and Crum-Cianflone 2010). In contrast to *Candida*,*Aspergillus* and *Cryptococcus* species, *Histoplasma* species more commonly cause infections in healthy individuals (Köhler *et al*. 2017). Nevertheless, a compromised innate as well as adaptive immune response increases the susceptibility to histoplasmosis (Akram and Koirala 2020).

Tissue-resident macrophages and monocyte-derived macrophages especially play an essential role against invasive candidiasis (Austermeier *et al*. 2020), whereas alveolar macrophages (AMs) are essential for clearance of fungi like *Aspergillus*, *Cryptococcus* or *Histoplasma* species that enter our body via the airways (Newman 2005; Xu and Shinohara 2017). Neutropenia is a common risk factor for aspergillosis and invasive candidiasis, showing the crucial role of neutrophils in antifungal host defense (Herbrecht *et al*. 2000). Dendritic cells (DCs) are crucial for activation of the adaptive immune system. Dysfunctions of the adaptive immune system like the reduced CD4^+^ T cell function in AIDS patients increase the susceptibility for infections with *C. albicans*, *A. fumigatus*, *C. neoformans* or *H. capsulatum* (van de Veerdonk and Netea 2010). Interestingly, this predisposition manifests as mucosal *C. albicans* infections, in particular oropharyngeal candidiasis (OPC), but systemic *C. albicans* infections are also observed under such conditions (Fidel 2011). This is believed to be closely connected to the crucial roles of T helper responses in orchestrating oral mucosal resistance to infection (Gaffen and Moutsopoulos 2020; Scheffold, Bacher and LeibundGut-Landmann 2020). Antifungal immunity in the brain is connected to microglia that are the resident macrophage-like cells of the central nervous system (CNS), which show strong responses to fungal species like *C. albicans* (Blasi *et al*. 1991) and *C. neoformans* (Barluzzi *et al*. 1998). The C-type lectin receptor signaling adaptor CARD9 is expressed by microglia cells and its deficiency is associated with fungal brain infections (Drummond and Lionakis 2019). NK cells also exhibit antifungal effects (Schmidt, Tramsen and Lehrnbecher 2017) and a delayed NK cell reconstitution (e.g. after allogeneic stem cell transplantation) is associated with a higher risk of invasive aspergillosis (Weiss *et al*. 2020).

### Antifungal effector functions during host defense against fungal pathogens

After the recognition of pathogen-associated molecular patterns (PAMPs) via pathogen recognition receptors (PRRs), supported by opsonization, innate immune cells mount responses to counteract the invading fungi. At early stages of infection, macrophages detect and engulf fungal pathogens (Gilbert, Wheeler and May 2014) (Fig. 2A). In addition, through the release of cytokines and chemokines they recruit and activate other immune cells. When neutrophils migrate to the site of infection, they act against fungal pathogens through phagocytosis, oxidative bursts and NETosis (Gazendam *et al*. 2016; Urban and Nett 2019). The production of reactive oxygen species (ROS) by phagocytes can kill fungal pathogens, like *C. albicans*, directly (Grondman *et al*. 2019) or impact LC3-mediated phagocytosis during defense against *A. fumigatus* (Sprenkeler, Gresnigt and van de Veerdonk 2016). This is highlighted, for example, by the fact that chronic granulomatous disease (CGD) patients, incapable of producing ROS, are highly susceptible to aspergillosis (Segal *et al*. 2000). DCs represent the bridge to activate the adaptive immune system via antigen processing and presentation to T-cells (LeibundGut-Landmann *et al*. 2007). T-cell differentiation can influence infection in different ways. Th1 cells augment the innate immune function through the release of IFNγ (Lionakis and Levitz 2018), which increases the microbicidal capacity of macrophages (Netea *et al*. 2015). Th17 cells release proinflammatory cytokines such as IL-17 and IL-22, which mediate recruitment of neutrophils and induce production of antimicrobial peptides (Khader, Gaffen and Kolls 2009; Conti *et al*. 2016) (Fig. 2A). The importance of these T-cell types for antifungal defense is evident in corresponding knock-out mice that have an increased susceptibility to disseminated *C. albicans* infections (Balish *et al*. 1998; Huang *et al*. 2004), but also show a striking susceptibility to mucosal infections. Th2 responses can result in a detrimental immune response, manifesting in a higher susceptibly to disseminated *C. albicans* infections (Haraguchi *et al*. 2010) or an aberrant immune response to *A. fumigatus* spores connected to allergic bronchopulmonary aspergillosis (ABPA) (Knutsen and Slavin 2011). T regulatory cells can suppress inflammatory responses and are highly beneficial to prevent immunopathology in the case of ABPA (Montagnoli *et al*. 2006), but also allow *C. albicans* persistence in the gastrointestinal tract (De Luca *et al*. 2007).

### *In vitro* models to study interactions between fungi and immune cells

The interactions between fungi and the different effector functions of the immune system can be easily studied *in vitro* using cell lines (Table S1A, Supporting Information) and primary immune cells (Table S1B, Supporting Information). Cell lines have the advantage of easy handling and provide highly reproducible results. The availability of many reporter cell lines and the possibilities to generate transgenic/knockout cell lines represent valuable resources that allow the study of highly conserved mechanisms in the immunology against fungal infections. Nevertheless, central cellular processes such as pyroptosis, apoptosis and autophagy are considerably different or modified in cancer cell lines. Over the past few years, the essential role of these processes in shaping antifungal immunity has become increasingly clear (Kanayama and Shinohara 2016; Sprenkeler, Gresnigt and van de Veerdonk 2016; Dominguez-Andres *et al*. 2017; Evans, Sundaramurthy and Frickel 2018; O'Meara and Cowen 2018; Gonçalves *et al*. 2020; Thak *et al*. 2020; Weerasinghe and Traven 2020). Therefore, primary cells offer the highest similarity to the physiological situation. Primary immune cells are commonly isolated from peripheral human blood. By density gradient centrifugation, peripheral blood mononuclear cells (PBMCs) can be separated from erythrocytes and granulocytes (Munoz and Leff 2006). An important aspect to consider when using primary cells is that strong donor variation and even seasonal differences can influence experimental outcomes (Ter Horst *et al*. 2016). However, genetic differences between donors can also be exploited to analyze the impact of specific genotypes on the antifungal immune response (Lionakis *et al*. 2013; Smeekens *et al*. 2013; Matzaraki *et al*. 2017; Gresnigt *et al*. 2018b; Jaeger *et al*. 2019a,b). In these functional genomic approaches, immune cells of large cohorts of volunteers are screened for variation in specific immunological effectors such as cytokine release, ROS release or fungal killing. After genotyping the donors, the results of immunological phenotypes can be stratified based on the corresponding genotype. This knowledge on the influence of common genetic variations on the antifungal host response can provide valuable information about the role of certain genes in antifungal host defense. Combined with genetic association studies, functional genomics can be used to validate the impact of identified variants on immune pathways and susceptibilities to infections. In this way, crucial roles have been identified for CX3CR1 and its role in host defense against of systemic candidiasis (Lionakis *et al*. 2013), as well as for the SIGLEC15 receptor in the susceptibility to vulvovaginal candidiasis (Jaeger *et al*. 2019b). Conversely, knowledge about genetic variations that influence critical antifungal host defense pathways can lead to the discovery of genetic susceptibilities. In this way NOD2 variants were found to increase resistance to invasive aspergillosis (Gresnigt *et al*. 2018b).

### Macrophages

Interactions between macrophages/macrophage-like cells and fungal pathogens have been studied using cell lines like J774A.1, RAW, Ana-1, U937, BV-2 and THP-1 (Table S1A, Supporting Information). Such cell lines allow the generation of reporter constructs that can be used to monitor the activation of specific immune pathways. In this way, the importance of RAB-GTPases for maturation of *C. albicans*-containing phagosomes has been demonstrated (Bain *et al*. 2014; Okai *et al*. 2015). Another option is the use of macrophages derived from murine bone marrow cells and differentiated *in vitro* (BMDMs) (Table S1B, Supporting Information). A major advantage of this approach is the possibility to isolate BMDMs from mice with different genetic backgrounds (e.g. gene knockout or transgenic mice), thus providing a toolbox to obtain in-depth knowledge about key players of the host immune response during fungal infections. Such cells from knockout mice have been widely used to investigate, for example, inflammasome activation in the response to *C. albicans* (Kasper *et al*. 2018),*C. neoformans* (Guo *et al*. 2014) and *A. fumigatus* (Karki *et al*. 2015). In addition to BMDMs, human monocyte-derived macrophages (MDMs) can be used for *in vitro* studies. In such experiments, monocytes are isolated from PBMCs or whole blood and can be differentiated *in vitro* into a wide range of functionally different MDMs (Xue *et al*. 2014). MDMs have been used in numerous studies to dissect not only cytokine release, inflammasome activation, oxidative burst, phagocytosis and phagosome maturation after confrontation with fungi but also escape and survival mechanisms of fungi during these interactions (Smith, Dixon and May 2015; Gresnigt *et al*. 2018b; Kasper *et al*. 2018; O'Meara *et al*. 2018; Friedrich *et al*. 2019) (Table S1B, Supporting Information).

However, undifferentiated monocytes are also used to investigate how these cells are differentially activated (Halder *et al*. 2016; Dominguez-Andres *et al*. 2017; Klassert *et al*. 2017; Camilli *et al*. 2018; Leonhardt *et al*. 2018). The stimulation of monocytes using PAMPs such as β-glucan can induce epigenetic reprogramming, which alters the response to secondary *C. albicans* stimulation (Quintin *et al*. 2012), a concept known as innate immune memory or ‘trained immunity’. In contrast, the response to *C. albicans* can also be hampered by the induction of innate immune tolerance by PAMPs such as lipopolysaccharide (Grondman *et al*. 2019). Over the past years it has become increasingly evident that cell metabolism is linked with immune cell functionality. Global as well as targeted profiling of metabolic pathways in primary immune cells, especially monocytes and macrophages, have been used to uncover immunometabolism in response to fungi (Dominguez-Andres *et al*. 2017; Gonçalves *et al*. 2020; Weerasinghe and Traven 2020).

Since pathogenic fungi often colonize and infect specific organs, the corresponding tissue macrophages offer the highest physiological relevance. For example, specific cells lines such as the murine alveolar macrophage cell lines MH-S (Mattern *et al*. 2015) and AMJ2-C11 (Pitangui Nde *et al*. 2015) are used to study fungal pathogens that cause pulmonary infections (Table S1A, Supporting Information). Alternatively, primary alveolar macrophages can be used to study the immune response of pulmonary fungal infections *ex vivo*. Though, the limited availability of these cells makes it challenging to obtain sufficient numbers for experiments. Nevertheless, protocols are available to obtain large numbers of AMs from bronchoalveolar lavage (BAL) (Busch *et al*. 2019) or resected lung tissue (Nayak *et al*. 2018). Similarly, peritoneal macrophages have been used to study the interactions with *Candida* spp. (Ifrim *et al*. 2016; Shimamura *et al*. 2019). Because peritoneal macrophages are easier to obtain in larger quantities than AMs, they have also been used for interaction studies with *H. capsulatum* (primarily infecting the lung) (Youseff *et al*. 2012; Huang *et al*. 2018; Shen *et al*. 2018) (Table S1B, Supporting Information). To dissect fungal interactions with immune cells in the brain, BV-2 microglia cells (Blasi *et al*. 1990) (Table S1A, Supporting Information) were co-cultured with astrocytes to demonstrate that candidalysin induces IL-1β release, which in turn mediates neutrophil recruitment (Drummond *et al*. 2019) (Fig. 1B).

Interaction studies with macrophages revealed mechanisms enabling fungal cells to evade macrophage phagocytosis or to escape from phagosomes. Masking of cell wall epitopes can prevent the detection of *A. fumigatus*, *C. albicans* and *H. capsulatum* by macrophages (Rappleye, Eissenberg and Goldman 2007; Aimanianda *et al*. 2009; Ballou *et al*. 2016). Morphological changes such as titan cell formation by *C. neoformans* (Okagaki and Nielsen 2012) or filamentation by *A. fumigatus* and *C. albicans* influence phagocytosis efficiency (Lewis *et al*. 2012; Erwig and Gow 2016; Maxson *et al*. 2018). Additionally, these fungi can inhibit phagosome acidification or phagosome maturation to prevent intracellular killing. These processes are reviewed in detail by Gilbert, Wheeler and May (2014) and Seider *et al*. (2010).

Irrespectively of the immune cell type used, numerous readouts are available to study interactions between fungi and cells of the immune system. Transcriptional profiling has provided indispensable insights into the interplay between immune cells and fungal pathogens. Specifically, dual-species transcriptional profiling has helped to elucidate key features of the adaptations of fungal cells in response to immune cells and *vice versa* (Niemiec *et al*. 2017; Munoz *et al*. 2019). Given the crucial role of phagocytes in fungal clearance, protocols established to investigate phagocytosis and phagosome maturation are common (Fig. 2A). Using live-cell microscopy, phagocytosis and viability dynamics can be studied on a kinetic scale involving multiple phagocytes (Smith, Dixon and May 2015; Gresnigt *et al*. 2018a; Kasper *et al*. 2018; Lim *et al*. 2018; Guimaraes *et al*. 2019; Seoane *et al*. 2020). For example, a struggle for glucose availability between macrophages and *C. albicans* was demonstrated to be crucial in dictating inflammasome activation (Tucey *et al*. 2020).*Candida albicans* cells however, can filament thereby complicating clearance through phagocytosis (Erwig and Gow 2016). Phagocytosis and phagosome maturation can also be examined in detail on a single-cell level (Bain *et al*. 2014; Okai *et al*. 2015; Westman *et al*. 2018). Such studies have contributed to the understanding of the role of phagosome–lysosome fusion in maintaining phagosome integrity while fungal cells filament inside the phagosome (Westman *et al*. 2020). Apart from live cell imaging, phagocytes can also be fixed at specific time-points to investigate the co-localization of proteins to the phagosome using immunofluorescence staining. In this way, LC3-associated phagocytosis has been investigated as a crucial pathway to improve phagocytosis efficiency of *H. capsulatum* and *A. fumigatus* (Huang *et al*. 2018; Kyrmizi *et al*. 2018). Using a similar approach, a key role has been shown for flotillin-dependent microdomains or lipid rafts in phagosome formation for efficient host defense against *A. fumigatus* (Schmidt *et al*. 2020).

### Natural killer (NK) cells

Primary NK cells can be obtained from PBMCs by different isolation kits (Wang *et al*. 2017). NK cells have been studied alone or in co-culture with other immune cells and have been observed to have direct antifungal capacity against *C. neoformans* through the release of perforins (Wiseman *et al*. 2007). The recognition of β1,3-glucan through the NKp30 receptor was identified to trigger and enhance the killing of *C. albicans* and *C. neoformans* by NK cells (Li *et al*. 2018). Other *in vitro* studies revealed an exhausted phenotype of NK cells, when they degranulate in contact with *A. fumigatus* (Santiago *et al*. 2018). NK cell activation in response to *Candida* species has been observed to occur indirectly by cross talk with monocytes (Marolda *et al*. 2020). Similarly, for *A. fumigatus*, crosstalk between NK cells and DCs was found to mediate DC activation (Weiss *et al*. 2018). Further, direct antifungal effects of NK-cells against *A. fumigatus* have been associated with release of IFNγ (Bouzani *et al*. 2011) (Table S1B, Supporting Information).

### Neutrophils

Using hypotonic lysis of erythrocytes or other gradient solutions like PolymorphPrep^©^ (Progen, Heidelberg, Germany) (Degel and Shokrani 2010), primary neutrophils can be isolated from PBMCs to investigate their interaction with fungi. Neutrophils can act as phagocytes, but can also form neutrophil extracellular traps (NETs) and release cytokines in the presence of fungal cells. These features were studied intensively *in vitro* (Urban *et al*. 2006; Bruns *et al*. 2010; Rocha *et al*. 2015; Sun and Shi 2016; Dasari *et al*. 2018; Thompson-Souza *et al*. 2020). By studying phagocytosis, killing, NETosis and cytokine release, spleen tyrosine kinase (Syk) was identified as a crucial mediator for inducing antifungal effector mechanisms against various *Candida* species (Negoro *et al*. 2020). Another aspect is to monitor how these phagocytes migrate to the site of infection. Chemotaxis assays using specialized *in vitro* systems (Richards *et al*. 2004; Chen 2005; Thunström Salzer *et al*. 2018) can be used to elucidate this process in the context of fungal infections (Coenjaerts *et al*. 2001; Drummond *et al*. 2015; Rieber *et al*. 2016) (Table S1B, Supporting Information). ROS release or oxidative bursts in response to fungal pathogens can be assessed not only in neutrophils (Boyle *et al*. 2011; Liu *et al*. 2018) but also in monocytes (Wellington, Dolan and Krysan 2009; Brunel *et al*. 2018) and macrophages (Wolf *et al*. 1987; Youseff *et al*. 2012; Sun *et al*. 2014; Arce Miranda *et al*. 2019) (Fig. 2A; Table S1B, Supporting Information). Using a modified model, in which *C. albicans* cells are grown in clusters on poly-l-lysine coated glass slides, neutrophils were observed to form ‘swarms’ to efficiently use oxidative stress mechanisms to attack *C. albicans* (Hopke *et al*. 2020).

### Dendritic cells, T-cells and whole blood models

Virtually all immune cell types are being employed to study transcriptional responses to fungal pathogens (Smeekens *et al*. 2013; Hellwig *et al*. 2016; Van Prooyen *et al*. 2016; Niemiec *et al*. 2017) as well as cytokine and chemokine responses (Coady and Sil 2015; Becker *et al*. 2016; Marischen *et al*. 2018) to fungal pathogens (Fig. 2A). Often such studies involve crosstalk between different immune cell types such as antigen-presenting cells and cells of the adaptive immune system. PBMCs are frequently used due to their composition of innate and adaptive immune cells and allow the study of innate host responses (Becker *et al*. 2016; Alvarez-Rueda *et al*. 2020), but also T-cell mediated responses such as Th1, Th17, Th2 and Tregs (Zielinski *et al*. 2012; Gresnigt *et al*. 2013; Becker *et al*. 2015; Raijmakers *et al*. 2017; Page *et al*. 2018; Vogel *et al*. 2018) (Fig. 2A). For example, using PBMCs, the type I interferon pathway was identified to play a crucial role in *C. albicans* defense (Smeekens *et al*. 2013). Interactions between DCs and T-cells were used to investigate how the adaptive immune response is polarized through antigen presentation, co-stimulation and the cytokine environment (van der Does *et al*. 2012; Stephen-Victor *et al*. 2017). DC maturation can be examined in transwell systems (Lother *et al*. 2014) or by profiling maturation features via flow cytometry (Pietrella *et al*. 2005; Hefter *et al*. 2017; Vivas *et al*. 2019). For interaction studies including a wide range of immune cell types, whole blood models were used to gain information about fungal killing (Hunniger *et al*. 2014), transcriptional responses (Dix *et al*. 2015; Kämmer *et al*. 2020), cytokine release (Oesterreicher, Eberl and Zeitlinger 2019) and platelet interactions (Fréalle *et al*. 2018; Eberl *et al*. 2019) (Table S1B, Supporting Information).

## STUDYING RESPIRATORY TRACT INFECTIONS WITH *ASPERGILLUS*, *HISTOPLASMA* AND *CRYPTOCOCCUS* SPP.

In the respiratory tract fungal pathogens such as *A. fumigatus*, *H. capsulatum* and *C. neoformans* can cause infections in predisposed hosts. Since the major biological niche of these fungi is the environment, fungal elements (mostly conidia or yeast) are frequently inhaled by the human host. The healthy immune system can clear these inhaled fungal elements, whereas immunocompromised individuals or patients with pre-existing pulmonary conditions may fail to clear fungi and have a higher risk to develop aspergillosis, histoplasmosis or cryptococcosis. The clinical manifestations of these fungal diseases, however, are very diverse. Infections with pathogenic *Aspergillus* species can develop differently, depending on the immune reaction and underlying lung pathology (Soubani and Chandrasekar 2002; van de Veerdonk *et al*. 2017). While a compromised immune response can result in invasive pulmonary aspergillosis, pre-existing lung injury can lead to the development of an aspergilloma and a chronic or hyper inflammatory response. Such responses can also provoke allergic bronchopulmonary aspergillosis (Kosmidis and Denning 2015). In immunocompromised patients, specifically patients suffering from AIDS, *C. neoformans* can cause either pulmonary cryptococcosis or can disseminate into other organs after an (asymptomatic) pulmonary infection (Setianingrum, Rautemaa-Richardson and Denning 2019). *Cryptococcusneoformans* cells can be engulfed by AMs and DCs and can survive within the phagolysosome, proliferate and eventually escape via non-lytic exocytosis (vomocytosis) (Fig. 2C I). Vomocytosis was also observed for *C. albicans* (Bain *et al*. 2012), *C. krusei* (García-Rodas *et al*. 2011), *A. nidulans* and *A. fumigatus* (Gresnigt *et al*. 2018a). Intracellular survival is one key strategy of *C. neoformans* to disseminate from the respiratory tract (Coelho, Bocca and Casadevall 2014). Other translocation routes involve fungal cells crossing the epithelial border via transcytosis (Fig.   2C II) or a direct migration through areas where the epithelial lining has been compromised (Fig. 2C III) (Denham and Brown 2018). *Histoplasma* cap*sulatum* can cause pulmonary histoplasmosis, and similar to *C. neoformans*, it can evade the immune system by hiding inside AMs (Ray and Rappleye 2019). Following growth and replication, it can induce apoptosis facilitating further dissemination within the bloodstream and lymphatic organs (Fig. 2C IV) (Long *et al*. 2003; Mihu and Nosanchuk 2012; Pitangui Nde *et al*. 2015). In contrast to *H. capsulatum* and *C. neoformans*, which grow as yeast during infection, *A. fumigatus* proliferates as hyphae in the lung, allowing deep tissue invasion (Fig. 2C V).

### Simple *in vitro* models mimicking lung infections

To mimic the alveolar environment, the pulmonary epithelial cell line A549, originating from a human alveolar cell carcinoma (Lieber *et al*. 1976), is frequently used to study pathogenicity attributes including adhesion (Gravelat *et al*. 2010; Pitangui *et al*. 2012; Teixeira *et al*. 2014), endocytosis (Liu *et al*. 2016), epithelial detachment (Kogan *et al*. 2004; Bertuzzi *et al*. 2014) and epithelial damage (Ejzykowicz *et al*. 2010; Bertuzzi *et al*. 2014). These studies revealed crucial roles for the *A. fumigatus* transcription factors PacC (Bertuzzi *et al*. 2014) and DvrA (Ejzykowicz *et al*. 2010) to mediate tissue invasion and damage. In addition, A549 cells were used to dissect pulmonary epithelial IL-8 responses to *C. neoformans* and *H. capsulatum* (Barbosa *et al*. 2007; Alcantara *et al*. 2020), and shed light on how different *A. fumigatus* isolates differentially regulate gene expression of epithelial cells (Watkins *et al*. 2018) (Table S2, Supporting Information). To examine the fungal translocation through the pulmonary epithelium, transwell models with different modifications have been employed (Fig. 1A).

### Complex *in vitro* models mimicking lung infections

Models that combine A549 cells with DCs (Morton *et al*. 2018) or a bilayer of human pulmonary artery endothelial cells (HPAECs) with (Morton *et al*. 2014) or without DCs (Hope *et al*. 2007; Belic *et al*. 2018) were utilized to model the cellular complexity in the alveolus and the cellular cytokine response to fungal infections. The translational capacity of such a model was reflected in a study that validated the measurement of galactomannan as a biomarker of fungal infection and antifungal efficacy *in* vitro (Hope *et al*. 2007). These models have also been employed for microscopy-based analyses, gene expression analysis and analysis of immune activation to gain insights into the host–*Aspergillus* interactions at the alveolar epithelial interface (Table S2, Supporting Information).

To more closely resemble the physiological situation, primary human bronchial or small airway epithelial (HBE, SAE) cells were used to study proinflammatory epithelial cytokine responses to *C. neoformans* infections (Guillot *et al*. 2008). These cells differentiate when cultured at an air-liquid interphase (ALI) into lung epithelium and were also used to assess the host response to *A. fumigatus* conidia. Transcriptome and proteome analyses revealed the upregulation of apoptosis, autophagy, translation and cell cycle pathways as well as the downregulation of complement and coagulation pathways (Toor *et al*. 2018). The combination of differentiated pulmonary epithelial cells with DCs and macrophages provides an even more complex model, which allows the study of the interplay between fungal cells, the epithelium and the immune system (Chandorkar *et al*. 2017). As an alternative strategy to investigate *Aspergillus* spp. infections, bronchial mucosal tissue resected from cancer patients was used. Using this *ex vivo* model, adhesion, invasion, damage and structural changes of the epithelium were investigated (Amitani and Kawanami 2009). Although the latter model represents human physiology, its applicability is limited by the difficulty of obtaining patient material. Besides confounding factors, such as therapies and medication, inter-individual differences may impact the validity of this model and the ability to obtain reproducible results.

### Lung-on-chip models

Most lung models used so far are cultured statically and thus are not subjected to shear stress. Further, these models rarely consider the impact of additional members of the microbial community, such as the lung microbiota in the infection process. A number of lung-on-chip models have been established that reflect additional physiological key features of the lung. A ‘breathing’ alveolus-on-chip is mimicked by stretching and contraction of a membrane using a vacuum, which leads to an increased uptake of nanoparticles of the epithelium and transport to the vasculature (Huh *et al*. 2010; Stucki *et al*. 2018). Mechanostimulation represents an important biophysical cue since the stretching of the lungs influences repair mechanisms in damaged epithelial cells and might also play a significant role during fungal invasion (Desai, Chapman and Waters 2008). Deinhardt-Emmer and colleagues established an alveolus-on-chip model that harbored immune cells and consisted of two compartments. In the upper compartment, lung epithelial cells differentiated into the two types of alveolar epithelial cells and were separated by a porous membrane from an endothelial lining, subjected to flow in the lower compartment (Deinhardt-Emmer *et al*. 2020) (Fig. 1A). Although this model was not used to dissect fungal–host interactions so far, it revealed new insights about the interplay of *S. aureus* and influenza virus at the alveolar–capillary interface. During co-infection, increased inflammatory responses were observed including cytokine expression and loss of barrier function similar to severe clinical outcomes of patients with bacterial-viral superinfections (Deinhardt-Emmer *et al*. 2020). Other platforms have used human alveolar epithelial cells (hAEpCs), and also integrated neutrophils (Huh *et al*. 2010; Benam *et al*. 2016; Jain *et al*. 2018; Zhang *et al*. 2018). Future models can be colonized with (additional) members of the pulmonary microbiome to investigate the interplay with fungi, which can contribute to progression of pulmonary fungal infections (Kolwijck and van de Veerdonk 2014). Taken together, current lung-on-chip models can produce a microenvironment resembling the *in vivo* physiology by imitating an ALI, mechanical strain and immune responses. This can facilitate the establishment of sophisticated pulmonary-infection models.

## STUDYING COLONIZATION AND INFECTION OF THE ORAL CAVITY, THE INTESTINAL TRACT AND VAGINAL TRACT BY *CANDIDA* SPP.

In the oral cavity, the intestinal-, and vaginal tract, *Candida* spp. normally live as harmless commensal yeasts. However, some opportunistic *Candida* spp. can cause infections. These range from mucocutaneous infections such as OPC (Millsop and Fazel 2016) and VVC (Rosati *et al*. 2020) to invasive candidiasis (Pappas *et al*. 2018). Diverse predispositions, like immunosuppression, an impaired barrier function and an imbalanced microbiota, are prerequisites to enable infection of *Candida* species. However, both predisposition and protection by an adjusted immune response differ between the specific types of infections. In the following sections we discuss *in vitro* models used to study *C. albicans* and *C. glabrata* interactions with the host in three different niches of the human body.

### Studying *Candida* spp. infections of the oral cavity

OPC occurs mostly in combination with the use of broad-spectrum antibiotic therapy and immune suppression, e.g. through HIV/AIDS, chemotherapy or radiation therapy. Further, neonates, diabetic and elderly individuals are more susceptible (Patil *et al*. 2015). *Candida albicans* is the most prevalent species, but also other *Candida* species like *C. glabrata*,*C. dubliniensis*,*C. krusei*,*C. kefyr*,*C. parapsilosis*,*C. stellatoidea* and *C. tropicalis* can be found in oral lesions (Millsop and Fazel 2016). *Candida albicans* mainly interacts with the oral epithelium by invading cells via active penetration (Fig. 2E I) and/or induced endocytosis (Fig. 2E II) (Phan *et al*. 2007; Dalle *et al*. 2010; Wachtler *et al*. 2011a; Sheppard and Filler 2014; Naglik *et al*. 2017), or invasion of the tissue by degradation of E-cadherin, thereby disrupting the epithelial barrier (Fig. 2E III) (Villar *et al*. 2007). *In vivo*, the uppermost layer of the oral epithelium consists of stratified squamous epithelium, followed by a basal membrane and fibroblasts in the lamina propria.

### Simple *in vitro* models mimicking oral infections

To study *Candida*–host interactions of the oral cavity, oral epithelial cells are commonly used. TR146 cells are derived from a squamous cell carcinoma of the buccal mucosa (Rupniak *et al*. 1985) and used to investigate invasion (Puri *et al*. 2019), damage (Wilson *et al*. 2014; Meir *et al*. 2018) and gene expression (Schaller *et al*. 1998; McCall, Kumar and Edgerton 2018; Meir *et al*. 2018). The TR146 model has contributed significantly to the understanding of *C. albicans* pathogenicity by showing that the peptide toxin candidalysin is responsible for the capacity of *C. albicans* hyphae to cause damage (Moyes *et al*. 2016). The same model was used to demonstrate that candidalysin also activates epithelial proinflammatory responses through the epithelial growth factor receptor (Ho *et al*. 2019) and its synergistic signaling with IL-17 (Verma *et al*. 2017). Immortalized oral mucosal cells (OKF6/TERT-2) (Dickson *et al*. 2000) have also been used to study epithelial transcriptional responses (Liu *et al*. 2015), to visualize *C. albicans* invasion (Wollert *et al*. 2012) and to demonstrate that invasion is, in part, mediated through endocytosis (Solis *et al*. 2017; Swidergall *et al*. 2018). The same cell line was used to show that damage is mediated through white cells in contrast to opaque cells (Solis *et al*. 2018). Furthermore, Epha2 was identified as an epithelial cell pattern recognition receptor for fungal β-glucans, activating a signal cascade that results in a proinflammatory and antifungal response (Swidergall *et al*. 2018).

Tongue cells derived from a squamous cell carcinoma (SCC15) represent a third cell type used to dissect interactions of *C. albicans* with the oral epithelium (Lindberg and Rheinwald 1990). Similar to the studies discussed above, SCC15 cells were used to investigate epithelial damage (Kumar *et al*. 2015), invasion (Villar *et al*. 2007) and cytokine release (Dongari-Bagtzoglou and Kashleva 2003) (Table S3A, Supporting Information).

### Complex *in vitro* models mimicking oral infections

In addition to monolayer models (Fig. 1A), organotypic 3D models known as reconstituted human oral epithelium (RHOE) are commonly used to study oral *Candida* spp. infections due to their histological similarity to physiological oral epithelium. In these RHOE models, TR146 cells are cultured on a polycarbonate filter at an ALI with culture medium on the basal side, resulting in a multilayer model with differentiated cells (Fig.   1B). This model has been used to study epithelial damage (Silva *et al*. 2011; Mailander-Sanchez *et al*. 2017) and fungal (Spiering *et al*. 2010) or host cell gene expression (Wagener, Mailander-Sanchez and Schaller 2012) (Table S3A, Supporting Information). In addition, the model was used to show enhanced invasion and tissue damage during co-infection of *C. albicans* and *C. glabrata* (Silva *et al*. 2011). Because fungal biofilm formation is crucial for the development of caries and OPC, the RHOE model has also been used to analyze the expression of *C. albicans* virulence genes associated with biofilm formation (Nailis *et al*. 2010). Similar RHOE models exist, containing collagen embedded fibroblasts from mice and oral mucosal cells OKF6/TERT-2 cells, differentiated at an ALI (Dongari-Bagtzoglou and Kashleva 2006a,b). Since the interplay with the oral microbiota plays an essential role for the maintenance of a commensal state of *C. albicans* or for development of OPC (Montelongo-Jauregui and Lopez-Ribot 2018), the organotypic 3D models were also used to study interactions between *C. albicans* and bacteria. For example, antagonistic interactions between *Lactobacillus rhamnosus* and *C. albicans* were dissected (Mailander-Sanchez *et al*. 2017). Furthermore, fungal-induced dysbiosis after chemotherapy (Bertolini *et al*. 2019) and synergistically increased tissue damage during interactions with *S. mutans* (Diaz *et al*. 2012) were observed. Additionally, biofilm formation of *C. albicans* and *C. glabrata* after chemotherapeutic treatment was examined in the latter organotypic 3D model (Sobue *et al*. 2018). The model was further ‘humanized’ by using human fibroblasts and spontaneously immortalized keratinocytes to analyze interactions between *C. albicans* and *S. aureus* (de Carvalho Dias *et al*. 2018) (Table S3A, Supporting Information).

### *In vitro* modeling of *C. albicans* stomatitis

*C. albicans* mediated stomatitis, an inflammatory reaction of the oral mucosa, is a major complication for users of removable dental prostheses, but also common in smokers or patients suffering from diabetes mellitus (Salerno *et al*. 2011; Javed *et al*. 2017; Alzayer *et al*. 2018). To model this oral infection, primary human palate epithelial cells (HPECs) were used to study the host response to *C. albicans* in terms of apoptosis, nitric oxide production (Casaroto *et al*. 2019) and mucosal gene expression (Offenbacher *et al*. 2019). Similarly, a combination of TR146 cells and primary fibroblasts was used for adhesion and gene expression studies (Morse *et al*. 2018) (Table S3A, Supporting Information).

### Mucosa-on-chip models

Monolayer or multilayered mucosal models commonly feature a perpendicular configuration. This vertical culture arrangement hampers the individual monitoring of different cell layers by microscopy, and resolution decreases in deeper layers. A horizontal organization of cell layers was applied in a mucosa-on-chip model (Rahimi *et al*. 2018) consisting of microchambers, which were aligned in parallel and interconnected by pores. A central subepithelial chamber harbored a collagen hydrogel with gingival fibroblasts, while keratinocytes were seeded into the pores connecting the luminal and subepithelial compartment. The luminal chamber can be microfluidically perfused to imitate saliva and saliva flow, which is an important contributor to epithelial barrier integrity. A further refinement for both static and microfluidic models can include an endothelial lining and immune cells such as dendritic Langerhans cells, which are almost exclusively found in stratified squamous epithelium and have been shown to react to *Candida* species (Upadhyay *et al*. 2013).

### Studying *Candida* spp. colonization of the intestinal tract and intestinal translocation

Both *C. albicans* and *C. glabrata* colonize the human intestinal tract (Hallen-Adams and Suhr 2017). The gut represents the main reservoir of fungi, especially *C. albicans*, that can cause disseminated and systemic infections (Gouba and Drancourt 2015). In these life-threatening infections, the fungus overcomes the intestinal epithelium, which forms a barrier between the intestinal lumen and the sterile tissues of the human body. During this process, termed translocation, the fungus employs several mechanisms including active penetration (Fig. 2F I), paracellular translocation (Fig. 2F II) or migration through the intestinal epithelial layer without damaging the host cells (Fig. 2F III) (Allert *et al*. 2018; Basmaciyan *et al*. 2019). Certain predispositions favor fungal overgrowth and translocation: antibiotics induce an imbalance of the microbiota and cytostatic therapy or abdominal surgery, which compromise the barrier function (Pfaller and Diekema 2007). To better understand the conditions that keep *C. albicans* commensal or drive the commensal-to-pathogen shift, the interactions between *C. albicans* and the intestinal barrier are studied extensively to find ways to prevent or reverse this shift (Kumamoto, Gresnigt and Hube 2020).

### Simple *in vitro* models mimicking intestinal infections

Monolayers of cell lines originating from colorectal adenocarcinomas are widely used (Fig. 1A). The most common cell lines are Caco-2 and HT-29. Caco-2 cells differentiate spontaneously into a polarized monolayer with characteristic villi and tight junctions after 12 days of culture (Fogh, Wright and Loveless 1977). These cells were used to demonstrate that damage to the intestinal epithelium induced by *C. albicans* relies on a combination of adhesion-mediated contact sensing, tissue invasion through hyphal extension and damage by the expression of pathogenicity factors (Wachtler *et al*. 2011a). Interactions with non-pathogenic yeast cells that can antagonize *C. albicans* pathogenicity were examined (Lohith and Anu-Appaiah 2018; Kunyeit *et al*. 2019). Furthermore, receptor signaling pathways (Mao *et al*. 2019), induction of defensins (Gacser *et al*. 2014), impact on tight junctions (Goyer *et al*. 2016) and the potential of epithelial cells to discriminate between yeast and hyphal morphologies (Schirbel *et al*. 2018) are processes that can be analyzed in this model. A subclone of the Caco-2 cell line, C2BBe1, was often used in *in vitro* systems due to its more homogeneous brush boarder expression (Peterson and Mooseker 1992). A model of C2BBe1 cells cultured in transwell systems (Fig.   1A) was instrumental to elucidate important virulence requirements of translocation through the epithelial barrier and revealed a key role for candidalysin by mediating necrotic cell damage that allowed transcellular translocation (Allert *et al*. 2018). Additionally, using this model, a MAPK/NFκB mediated epithelial response to *C. albicans* infection was shown to increase epithelial resistance (Bohringer *et al*. 2016) (Table S3B, Supporting Information).

Essential features of *C. albicans* pathogenicity like adhesion, invasion and damage were also studied using the HT-29 cell line (Deng *et al*. 2015; Garcia *et al*. 2018). A methotrexate treatment of HT-29 cells, transformed these cells into mucus-secreting goblet cells (HT-29-MTX) (Lesuffleur *et al*. 1990). These mucus-secreting cells were instrumental in demonstrating the role of mucus in suppressing virulence-associated attributes of *C. albicans*, such as hypha formation (Kavanaugh *et al*. 2014).

### Complex *in vitro* models mimicking intestinal infections

As the intestinal epithelium consists of a myriad of cell types, combinations of different cell lines have been employed to more accurately mimic the *in vivo* situation. For example, a combination of Caco-2 cells and Raji B cells (human Burkitt's lymphoma) was used to study the interaction of *C. albicans* with an epithelial barrier including M-cells, which demonstrated M-cells as a preferred cell type for translocation via induced endocytosis (Fig. 2F IV) (Albac *et al*. 2016). In general, most *in vitro* models investigate *C. albicans* in its pathogenic state. To limit the pathogenicity of *C. albicans* and mimic commensalism, a mixture of C2BBe1 cells and the mucus-producing HT-29-MTX cells were colonized with *L. rhamnosus* to establish a basic ‘commensal’ model (Fig.   1B). Using this model, a damage reduction was observed in the presence of mucus and bacteria, both antagonizing *C. albicans* pathogenicity by reducing filamentation, proliferation and inducing shedding that physically separates hyphae from host cells (Graf *et al*. 2019) (Table S3B, Supporting Information).

### Intestine-on-chip models

Although 2D intestinal models mimic the fundamental physiological structures of the intestinal tissue such as mucus production, M-cells and brush border epithelium, they do not reflect the unique 3D architecture of the intestinal epithelial tissue consisting of villi and crypts. Cells in these models are cultured statically and are not subjected to the peristaltic movement characteristic for the intestine. In addition, *in vitro* models often lack immune cells, which convey tolerance towards commensals and trigger inflammatory responses when pathogens inflict damage to the intestinal lining. A number of intestine-on-chip models have been developed that recapitulate some of these key physiological features (Bein *et al*. 2018). In these models, Caco-2 cells grow out and form villi-like structures when grown on a membrane and exposed to shear stress (Kim and Ingber 2013). Microfluidic intestine models often include endothelial cells adjacent to epithelial cells in an individually perfused compartment. The luminal and the vascular compartment are separated by a porous membrane to facilitate transmigration of cells and cell communication. Innate immune cells such as monocytes can be implemented in the endothelial layer and differentiated into macrophages and DC-like cells, which tolerate inflammatory triggers in the intestinal lumen, but elicit a strong inflammatory response when a systemic infection is mimicked (Maurer *et al*. 2019). In this model, *C. albicans* invasion of the epithelial layer and subsequent invasion of the bloodstream compartment in the presence and absence of the commensal bacterium *L. rhamnosus* were investigated. Patient-derived colon epithelial cells are difficult to access, but can sufficiently be maintained in microfluidic platforms and produce a mucus layer resembling the *in vivo* thickness (Sontheimer-Phelps *et al*. 2020). 3D intestine-on-chip models will be valuable tools to uncover the role of commensals and their products, as well as host immune responses in the yeast-to-hypha transition of *C. albicans* in the future (Table S3B, Supporting Information).

### Intestinal organoids

Apart from intestine-on-chip models, human intestinal organoids have emerged as a valuable disease-modeling tool. Human intestinal organoids can be grown from adult stem cells extracted from intestine biopsies or induced pluripotent stem cells (iPSCs) (Rahmani *et al*. 2019) to form 3D organotypic structures by self-organization and resemblance of key embryonic signaling *in vitro* (Clevers 2016) (Fig. 1A). Intestinal organoids show a villus and crypt-like architecture with epithelial cells facing inwards, creating a lumen as an enclosed space (Sato *et al*. 2009; Spence *et al*. 2011). Organoid models face similar challenges like OOC platforms, such as additional cell types, immune cells, endothelial cells and extracellular matrix components that need to be incorporated to create a physiological microenvironment for cell differentiation and tissue development. However, mesenchymal cells and neural crest cells have already been successfully implemented in these models (Workman *et al*. 2017). Unlike microfluidic OOC models, stem cell-derived organoids currently lack perfusion and therefore deprive epithelial cells of shear stress and removal of metabolites. An idea has emerged that aims at combining self-assembling organoids with microfluidic OOC techniques, termed ‘Organoids-on-a-Chip’ (Park, Georgescu and Huh 2019) (Fig. 1A). The technique encompasses the maturation of organoids within a dynamic culture environment allowing the control of nutrient supply, establishment of biochemical gradients vital for self-organization of the organoids and the introduction of additional cell types.

### Studying *Candida* spp. infections of the vaginal mucosa

The vaginal mucosa represents another commensal niche of *Candida* spp. in the human body. VVC affects 70–75% of women in their reproductive age (Sobel 2007). Antibiotic treatment is a strong predisposing factor for VVC (Shukla and Sobel 2019), most likely due to the induced dysbiosis of the vaginal microbiome. *C*.*albicans* is the most prominent species isolated from VVC, followed by *C. glabrata* (Makanjuola, Bongomin and Fayemiwo 2018). The interactions between *Candida* spp. and the vaginal epithelium, as well as the vaginal microbiota, are complex (Pekmezovic *et al*. 2019; Kalia, Singh and Kaur 2020), and invasion of the epithelium occurs through active penetration (Fig. 2G I) and induced endocytosis (Fig. 2G II), while neutrophils are attracted simultaneously.

### Simple *in vitro* models mimicking vaginal infections

The VK2/E6E7 cell line originates from healthy human vaginal mucosal tissue and was immortalized by retroviral transduction (Fichorova, Rheinwald and Anderson 1997). This cell line was used to demonstrate synergistic interactions between *C. albicans* and streptococci (Pidwill *et al*. 2018) and a role for autophagy machinery in the survival of epithelial cells during *C. albicans* infection (Shroff and Reddy 2018). In addition, Type-I IFN signaling was elucidated to increase resistance of the epithelium to *C. albicans* infection (Li *et al*. 2017). By introducing high glucose conditions, this model has been used to demonstrate that the association of VVC in diabetes patients might be related to increased adhesion of *C. albicans* through a potential interaction with ICAM-1 (Mikamo *et al*. 2018). Another cell line, A431, originates from a vaginal epidermoid carcinoma. This cell line was used to investigate inflammatory cytokine responses and damage of A431 cells induced by candidalysin (Richardson *et al*. 2018). Additionally, the cell line was utilized to evaluate the impact of azole antifungal treatment on damage induced by *C. albicans* spp. (Wachtler, Wilson and Hube 2011b) (Table S3C, Supporting Information).

### Complex *in vitro* models mimicking vaginal infections

A reconstituted vaginal epithelium (RHVE) is available as an alternative model. RHVE is based on A431 cells, cultivated at an ALI, similar to the previously described RHOE (Fig. 1B). RHVE was used to demonstrate that *C. albicans* facilitates interactions of *C. glabrata* with the vaginal epithelium by increasing fungal colonization, invasion and damage of epithelial cells during co-infection (Alves *et al*. 2014). Furthermore, the adaptation of *C. glabrata* to an acidic vaginal environment was investigated using RHVE (Bernardo *et al*. 2017) (Table S3C, Supporting Information).

### Organ-on-chip models mimicking vaginal infections

Several OOC models for the female reproductive tract are available, predominantly to mimic the physiology of the endometrium, the uterus or the placenta (Mancini and Pensabene 2019). Possible OOC models of the vaginal mucosa should comprise stratified squamous epithelium and perfused endothelial cells, separated by a porous membrane. Immune cells can easily be integrated to recapitulate relevant inflammatory responses during hyphal invasion of the epithelium such as neutrophil recruitment.

*In vivo*, the vaginal tract harbors a microbiota that consist to a large extent of *Lactobacillus* species. Although predicted, it is not entirely clear whether the microbiota actually has a protective effect against *Candida* spp. infection and if so, whether diversity among microbial communities leads to a higher degree of protection (Cassone 2015).

## STUDYING FUNGAL BLOODSTREAM INFECTION AND CROSSING OF THE BBB

### Vascular infection models

Fungal dissemination into the bloodstream is a major driver for the development of multi-organ infections or sepsis. *Aspergillus fumigatus*, *H. capsulatum* and *C. neoformans* can enter the bloodstream after crossing the pulmonary alveolar epithelium (Fig. 2C), whereas *C. albicans* reaches the bloodstream mostly via the intestinal tract (Fig. 2F). Central venous catheters, surgery and parenteral nutrition represent additional entry routes, especially for *Candida* species (Hashemi Fesharaki *et al*. 2018). To exit the blood circulation and invade other organs, fungi interact with the endothelial lining of the blood vessels (Fig. 2D), which can be simulated by human umbilical vein endothelial cells (HUVECs) (Jaffe *et al*. 1973). Although access to umbilical cords is limited, high amounts of cells can be isolated from a single umbilical cord and stored frozen for several experiments (Crampton, Davis and Hughes 2007). HUVECs were used to dissect *C. albicans* adhesion to the endothelial lining (Fig.   2D I), for example, it was shown that a certain hyphal length is crucial for adhesion in a circulatory *in vitro* model that simulated physiological capillary blood pressure (Wilson and Hube 2010) (Fig. 1B). Following adhesion, three mechanisms to pass the endothelial barrier were discovered. Attached *Candida* cells can be endocytosed by endothelial cells (Phan *et al*. 2005; Liu *et al*. 2016) (Fig. 2D II), a process that depends on a complex formation including endothelial cell septin 7 (SEP7) and *N*-cadherin (Phan *et al*. 2013). Endocytosis was also described for *A. fumigatus*, independent of its morphology (Kamai *et al*. 2006) (Fig. 2D II). In addition, *Candida* spp. can cross the endothelial barrier via paracellular translocation (Fig. 2D III) or via leucocytes following engulfment (Fig. 2D IV) (Filler and Sheppard 2006; Grubb *et al*. 2008). It is likely that similar Trojan horse transport mechanisms following engulfment by mononuclear cells are exploited by intracellularly persistent *H. capsulatum* (Gilbert, Wheeler and May 2014) (Fig. 2D IV) as it already has been shown for *C. neoformans* (Coelho *et al*. 2019).

The ability of different *C. albicans* mutants to damage HUVECs was leveraged to identify virulence factors that are important for fungal dissemination (Sanchez *et al*. 2004). Similarly, the transcription factor DvrA was identified as crucial for endothelial damage induced by *A. fumigatus* (Ejzykowicz *et al*. 2010). Besides, the proteome profile of HUVECs was investigated during infection with *A. fumigatus* (Neves *et al*. 2017) and *C. neoformans* (Wang *et al*. 2011), indicating alterations that contribute to fungal invasion. Transcriptional profiling of HUVECs revealed the upregulation of genes involved in chemotaxis, stress response, angiogenesis and inhibition of apoptosis in response to *C. albicans* (Barker *et al*. 2008). A proinflammatory immune response associated with the release of TNF in HUVECs was reported after infections with *C. albicans* (Orozco, Zhou and Filler 2000) and *A. fumigatus* (Kamai *et al*. 2009; Neves *et al*. 2017). In addition, it was shown that neutrophils protect endothelial cells against *C. albicans*-induced damage in a co-culture model with HUVECs and neutrophils (Edwards *et al*. 1987) (Table S4, Supporting Information).

### Blood–brain barrier

Whereas cerebral infections with *Candida* spp. (Drummond *et al*. 2015), *Aspergillus* spp. (Rieber *et al*. 2016) or *Histoplasma* spp. (Schestatsky *et al*. 2006) are rare, meningitis is the most prominent complication during cryptococcosis (Srikanta, Santiago-Tirado and Doering 2014). Cerebral infections are induced when fungi cross the BBB, a part of the neurovascular unit (NVU). Other than the endothelial lining, the NVU consists of pericytes, forming a scaffold for endothelial cells together with the basal lamina. Endfeet of astrocytes provide a connection to neurons and microglia (van der Helm *et al*. 2016). A physical barrier between the blood circulation and the brain tissue is maintained by an intact NVU via zona occludens proteins and claudins.

### Simple *in vitro* models mimicking the BBB

Immortalized human brain vascular endothelial cells (HBMEC and HCMEC/D3) are commonly used for BBB models, whereas primary cells are not frequently used due to insufficient availability and loss of phenotype during culturing (Oddo *et al*. 2019). The HBMEC and HCMEC/D3 cell lines are especially suitable to model the BBB because of their expression of tight junction proteins, receptors and transporters (Weksler, Romero and Couraud 2013; Oddo *et al*. 2019). They can be cultured as monolayers on transwell inserts or cell culture plates and infected with *C. albicans* (Jong *et al*. 2001), *A. fumigatus* (Patel *et al*. 2018) or *C. neoformans* (Aaron *et al*. 2018) and used for transcytosis (Aaron *et al*. 2018), gene expression (Lahiri *et al*. 2019) and barrier integrity studies (Patel *et al*. 2018). For example, it was demonstrated that *C. neoformans* and *C. albicans* can pass the BBB via transcytosis (Fig. 2B I). True hyphae of *C. albicans* are associated with endocytosis by endothelial cells (Liu *et al*. 2011) (Fig. 2B I). *Cryptococcus neoformans*, however, was shown to also translocate paracellularly (Fig. 2B II) and use macrophages as a shuttle to cross the BBB using the Trojan horse mechanism mentioned above (Charlier *et al*. 2009; Santiago-Tirado *et al*. 2017) (Fig. 2B III). This mechanism was visualized and analyzed in detail using a co-culture model of HCMEC/D3 cells and THP-1 cells or primary monocytes (He *et al*. 2016; Santiago-Tirado *et al*. 2017) (Table S4, Supporting Information).

### BBB-on-chip models

2D transwell models of the BBB can be valuable tools to gain insights into how fungi invade the CNS. However, current models lack some key properties of the NVU. For example, endothelial cells need to experience shear stress to trigger the establishment of a barrier that limits Na^+^ and Cl^−^ ions efflux and influx (Oddo *et al*. 2019). Furthermore, to mimic the physiological situation more closely, the model should contain multiple cell types of the NVU such as astrocytes, pericytes and neurons since their communication influences each other's growth, differentiation and permeability (Abbott, Ronnback and Hansson 2006). A range of microfluidic BBB-on-Chip models has recently been developed, recapitulating the blood flow by perfusion of the endothelium in realistic dimensions and geometry and integration of various NVU cell types (Griep *et al*. 2013; Raasch *et al*. 2016; Maoz *et al*. 2018). In models using one cell type, HUVECs in astrocyte-conditioned medium or HCMEC/D3 cells have been cultured in a single perfused channel (Yeon *et al*. 2012; Griep *et al*. 2013; Englert *et al*. 2016). Using a CNS angiogenesis model comprising endothelial cells, pericytes, astrocytes and lung fibroblasts, it was demonstrated that a low vascular permeability can be achieved by co-culturing the different NVU cell types (Lee *et al*. 2020). These microfluidic BBB models can contribute to investigating the role of additional cell types of the NVU and shear stress in the transmigration of fungi across the BBB. Moreover, the implementation of innate immune cells would enable the simulation of inflammatory responses in the brain tissue following fungal invasion (Table S4, Supporting Information).

## FUTURE DIRECTIONS

### Interconnecting organ-on-chip systems to study fungal dissemination

Although the multiple infection models reviewed here have been and will be very useful tools to study fungal infections, we can expect a new generation of complex *in vitro* system based on OOC platforms. In fact, individual OOC systems can be combined to recapitulate multi-organ cross communication in an enclosed microfluidic network (Luni, Serena and Elvassore 2014). These platforms have the potential to investigate fungal infections not only at a single-organ level, but also at the multi-organ level, including systemic immune responses (Fig. 1A). The complexity of systemic immune reactions was only addressed in animal models until recently. Multi-organ-on-chip (MOC) models expand the toolbox with systems having a purely human genetic background to circumvent the problem of interspecies transferability. A range of MOC platforms have been developed that connect two or more organs such as the liver and intestine (Zhang *et al*. 2009; Chen, Miller and Shuler 2018; Ramme *et al*. 2019). MOC models provide the opportunity to study the dissemination of fungi throughout the body. It will allow (to mimic) tracking dissemination of *Candida* spp. from the intestine to the liver and kidney, the key target organs of disseminated candidiasis (Lionakis *et al*. 2011), or dissemination of *A. fumigatus*, *C. neoformans* and *H. capsulatum* from the lung to the brain, which has not been possible *in vitro* so far. An additional aspect to be elucidated using MOC models is the relationship between dysbiosis in the intestine resulting in overgrowth of *C. albicans* and concomitant biochemical changes in the brain or the liver (gut–brain axis and gut–liver axis, respectively) (Burrus 2012; Yang *et al*. 2017). However, MOC systems are still in their infancy and there are many obstacles to overcome. A current challenge is to scale the organs to their relative physiological size (Lee and Sung 2017; Rogal, Probst and Loskill 2017). Current MOC systems are mostly used for toxicity screening of drugs and chemicals and are constructed in a way to be suitable for this particular application (Rogal, Probst and Loskill 2017). MOC models dedicated for fungal studies may take into account other criteria, e.g. the distance between distinct tissues, the number of integrated immune cells, and possibilities to prevent adherence of fungi to tubing and subsequent clogging, to be applicable as tools.

### Human induced pluripotent stem cells as another cell source for fungal *in vitro* systems

The *in vitro* models discussed in this review rely on primary cells and cell lines. Human induced pluripotent stem cells (hiPSC) are an alternative source of cells and are highly relevant for biomedical research (Raasch *et al*. 2019). hiPSC can be generated by reprogramming adult tissue cells, such as fibroblasts, to an embryonic-like pluripotent state (Takahashi and Yamanaka 2006). Once reprogrammed, they can be differentiated into virtually all cell types except extra-embryonic cell types. Therefore, they offer the opportunity to establish OOC systems containing various cell types originating from a single donor. However, current models often combine hiPSC with primary cells and cell lines. Taking the BBB as an example, Brown and colleagues cultured HBMEC, glutamatergic neurons differentiated from iPSC, primary pericytes and astrocytes in a two-chamber model. The resulting system consisted of a brain compartment, which is separated from perfused vasculature by a porous membrane (Brown *et al*. 2015).

hiPSC are also utilized for the establishment of ‘patient-on-chip’ models to mimic genetic predispositions. Aspergillosis is a common complication of patients suffering from asthma and cystic fibrosis (CF) (Knutsen and Slavin 2011) or CGD (Leiding and Holland 1993); CARD9 and STAT1 mutations predispose for *C. albicans* CNS (Drummond *et al*. 2019) and mucocutaneous infections (van de Veerdonk *et al*. 2011), respectively, and diabetes mellitus is a common predisposition for histoplasmosis (Lockhart and Guarner 2019). Furthermore, intestinal fungi have been tightly connected to inflammatory bowel diseases (Leonardi, Li and Iliev 2018). Future OOC models might be able to reflect these predispositions by implementing hiPSC generated from patients bearing these diseases. Alternatively, specific mutations associated with the disease can be reproduced in hiPSC. For example, they have been successfully differentiated into macrophages and lung epithelial cells that carry mutations associated with CF (Pollard and Pollard 2018) and CGD (Brault *et al*. 2017). Although there has been substantial progress in OOC systems incorporating hiPSC, caution should be exercised: Protocols for differentiation require optimization and standardization, especially the understanding of factors promoting differentiation needs improvement. Differentiation might differ under static and dynamic conditions (Luni, Serena and Elvassore 2014; Rogal, Probst and Loskill 2017). Standardization of these aspects is crucial to guarantee reproducibility of findings from different labs.

## CONCLUDING REMARKS

To study human fungal infections on a higher level of complexity, expertise of fungal infection biology and the OOC platforms needs to be combined. This will ensure studies in the most suitable *in vitro* model, providing conditions akin to the *in vivo* situation. For example, 3D intestine-on-chip models will be valuable tools to uncover the role of microbial commensals and their products, as well as the host immune responses to a local yeast-to-hypha transition of *C. albicans*. In the future, it would be favorable to make use of experience gained with MOC systems to mimic and follow fungal dissemination throughout the body and evaluate novel therapeutic strategies addressing fungal infections.

## ACKNOWLEDGEMENT

We thank Jakob Sprague for critical reading of the manuscript.

## Supplementary Material

fuab005_Supplemental_File
