# Supplementary material for: In vitro infection models to study fungal–host interactions
Source: FEMS Microbiol Rev. 2021 Feb 1;45(5):fuab005. doi: 10.1093/femsre/fuab005 (PMC8498566; doi:10.1093/femsre/fuab005)
Supplement: fuab005_Supplemental_File [file fuab005_supplemental_file.docx]

**Table S1: Overview about the different immune cell *in vitro* models, their structure, possible read outs and applications for the different fungi**

| Model | Structure | Readout | Reference |
| --- | --- | --- | --- |
| 1. **Macrophage (like) cell lines** | | | |
| **J774A.1**  (Mouse cell line) | Monoculture | Fungal killing | *A. fumigatus*: (Gresnigt *et al.* 2018a) |
|  |  | Phagocytosis | *A. fumigatus*: (Gresnigt *et al.* 2018a)  *C. albicans*: (Cottier *et al.* 2019; Duvenage *et al.* 2019; Loureiro *et al.* 2019; Rudkin *et al.* 2018)  *C. neoformans*: (Bryan *et al.* 2014) |
|  |  | Cytokine release | *A. fumigatus:* (Cho *et al.* 2016) |
| **RAW**  (Mouse cell line) | Monoculture | Fungal killing | *C. glabrata:* (Chew *et al.* 2019) |
|  |  | Proteomics | *A. fumigatus*: (Schmidt *et al.* 2018) |
|  |  | ROS production | *C. albicans*: (Arce Miranda *et al.* 2019) |
|  |  | Phagosome maturation | *C. albicans:* (Bain *et al.* 2014; Okai *et al.* 2015) |
| **Ana-1**  (Mouse cell line) | Monoculture | Apoptosis | *C. albicans*: (Jiang *et al.* 2019) |
| **THP-1**  (Human cell line) | Monoculture | Phagocytosis | *C. albicans* : (Liu *et al.* 2019; Vaz *et al.* 2019) |
|  |  | Proteomics | *C. albicans*: (Vaz *et al.* 2019) |
|  |  | Cytokine release | *A. fumigatus:* (Oya *et al.* 2019)  *C. albicans*: (de Albuquerque *et al.* 2018) |
|  |  | ROS release | *A. fumigatus:* (Sun *et al.* 2014) |
| **U937**  (Human cell line) | Monoculture | Adhesion | *C. albicans:* (Lopez et al. 2014)  *C. glabrata:* (Kuhn and Vyas 2012) |
|  |  | Internalization | *H. capsulatum:* (Scott and Woods 2000) |
|  |  | Cytokine release | *C. albicans:* (Kaya et al. 2011) |
| **MH-S**  (Mouse alveolar macrophage cell line) | Monoculture | Phagocytosis | *A. fumigatus:* (Mattern *et al.* 2015) |
| **AMJ2-C11**  (Mouse alveolar macrophage cell line) | Monoculture | Intracellular yeast arrangement | *H. capsulatum:* (Pitangui Nde et al., 2015) |
| **BV-2**  (Mouse microglial cell line) | Monoculture | Cytokine release | *C. albicans:* (Wu et al. 2019b)  *C. neoformans*: (Barluzzi *et al.* 1998) |
| **BV-2**  (Mouse cell line)  **+**  **C8-D1A Astrocytes**  (Mouse cell line) | Co-Culture | Cytokine release | *C. albicans:* (Drummond et al. 2019) |
| 1. **Primary cells** | | | |
| **Bone marrow derived macrophages**  **BMDMs**  (Murine primary cells) | Monoculture | Phagocytosis | *A. fumigatus:* (Gresnigt *et al.* 2018a)  *C. albicans:* (Haider *et al.* 2019; Okai *et al.* 2015)  *H. capsulatum*: (Baltazar *et al.* 2018; Guimaraes *et al.* 2019) |
|  |  | Cytokine release | *A. fumigatus*: (Rubino *et al.* 2012)  *C. albicans*: (Alsina-Beauchamp *et al.* 2018; Thompson *et al.* 2019; Wang *et al.* 2019)  *C. neoformans*: (Veloso Júnior *et al.* 2019) |
|  |  | Exocytosis | *C. neoformans*: (Stukes and Casadevall 2014) |
|  |  | Transcriptomics | *C. albicans:* (Muñoz et al. 2019) |
| **Alveolar macrophages**  (Murine/Human primary cells) | Monoculture | Phagocytosis | *A. fumigatus:* (Grimm *et al.* 2014; Wu *et al.* 2016)  *C. neoformans*: (Hansakon *et al.* 2019; Walsh *et al.* 2017)  *H. capsulatum*: (Pereira *et al.* 2018; Tagliari *et al.* 2012) |
|  |  | Autophagy | *A. fumigatus:* (Dai *et al.* 2018) |
|  |  | Apoptosis | *H. capsulatum:* (Deepe and Buesing 2012) |
|  |  | Cytokine release | *A. fumigatus:* (Zhang *et al.* 2017a)  *H. capsulatum*: (Coady and Sil 2015) |
| **Peritoneal macrophages**  (Murine primary cells) | Monoculture | Phagocytosis | *H. capsulatum*: (Huang et al., 2018) |
|  |  | Autophagy | *C. albicans:* (Ifrim et al. 2016)  *C. glabrata*: (Shimamura *et al.* 2019) |
|  |  | Cytokine release | *H. capsulatum*: (Shen *et al.* 2018) |
|  |  | ROS release | *H. capsulatum:* (Youseff *et al.* 2012) |
| **Monocyte derived macrophages**  **MDMs**  (Human primary cells) | Monoculture | Phagocytosis | *C. albicans:* (Behrens *et al.* 2019; Munawara *et al.* 2017) |
|  |  | Phagosome maturation | *C. neoformans*: (Smith *et al.* 2015) |
|  |  | Cytokine release | *A. fumigatus:* (Gresnigt *et al.* 2018b)  *C. albicans:* (Kasper *et al.* 2018; O'Meara *et al.* 2018)  *H. capsulatum*: (Friedrich *et al.* 2019) |
|  |  | ROS release | *A. fumigatus:* (Gresnigt *et al.* 2018b)  *H. capsulatum:* (Wolf *et al.* 1987) |
|  |  | Immuno-metabolism | *A. fumigatus:* (Gonçalves et al. 2020) |
| **Monocytes**  (Human primary cells) | Monoculture | Phagocytosis | *A. fumigatus:* (Brunel *et al.* 2017; Kyrmizi *et al.* 2018)  *C. albicans:* (Camilli *et al.* 2018; Halder *et al.* 2016)  *C. neoformans*: (Charlier *et al.* 2009) |
|  |  | Metabolomics | *C. albicans:* (Grondman *et al.* 2019) |
|  |  | Cytokine release | *C. albicans:* (Dominguez-Andres *et al.* 2017; Leonhardt *et al.* 2018) |
|  |  | ROS release | *A. fumigatus:* (Brunel *et al.* 2018)  *C. albicans*: (Camilli *et al.* 2018; Wellington *et al.* 2009) |
| **Monocytes**  (Human primary cells)  **+**  **DCs**  (Human primary differentiated cells) | Co-culture | Phagocytosis  Cytokine release | *C. neoformans*: (Alvarez *et al.* 2009; Kelly *et al.* 2005) |
| **Neutrophils**  (Human primary cells) | Monoculture | Fungal killing | *A. fumigatus*: (Dasari *et al.* 2018; Gazendam *et al.* 2016; Jones *et al.* 2019)  *C. albicans*: (Essig *et al.* 2015; Gazendam *et al.* 2016; Jones *et al.* 2019; Salvatori *et al.* 2018) |
|  |  | Phagocytosis | *A. fumigatus:* (Brunel *et al.* 2017)  *C. neoformans*: (Sun *et al.* 2016) |
|  |  | NET formation | *A. fumigatus*: (Bruns *et al.* 2010; Clark *et al.* 2018; Ellett *et al.* 2017; Röhm *et al.* 2014; Silva *et al.* 2019)  *C. albicans*: (Campos-Garcia *et al.* 2019; Guiducci *et al.* 2018; Johnson *et al.* 2017; Negoro *et al.* 2020; Urban *et al.* 2006; Wu *et al.* 2019a; Zawrotniak *et al.* 2019)  *C. neoformans*: (Rocha *et al.* 2015)  *H. capsulatum*: (Thompson-Souza *et al.* 2020) |
|  |  | Transcriptomics | *C. albicans*: (Niemiec *et al.* 2017) |
|  |  | ROS release | *A. fumigatus:* (Boyle *et al.* 2011)  *C. albicans:* (Liu *et al.* 2018; Miramón *et al.* 2012; Salvatori *et al.* 2018) |
|  | Monoculture on poly-L-lysine coated glass slides | Swarming | *C. albicans:* (Hopke et al. 2020) |
|  | Monoculture on Transwell  (top: neutrophils  Bottom: chemoattractant) | Chemotaxis | *A. fumigatus:* (Rieber *et al.* 2016)  *C. albicans*: (Drummond *et al.* 2015)  *C. neoformans:* (Coenjaerts *et al.* 2001) |
| **Dendritic cells**  **DCs**  (Human primary differentiated cells) | Monoculture | Phagocytosis | *A. fumigatus*: (Lother *et al.* 2014)  *H. capsulatum*: (Gildea *et al.* 2001; Nguyen *et al.* 2018) |
|  |  | Maturation | *A. fumigatus*: (Fliesser *et al.* 2016; Hefter *et al.* 2017; Lother *et al.* 2014)  *C. albicans:* (Roudbary *et al.* 2009; Vivas *et al.* 2019)  *C. glabrata:* (Bazan *et al.* 2018)  *C. neoformans:* (Pietrella *et al.* 2005) |
|  |  | Transcriptomics | *A. fumigatus*: (Srivastava *et al.* 2019)  *H. capsulatum*: (Van Prooyen *et al.* 2016) |
|  |  | Cytokine release | *C. albicans*: (Maher *et al.* 2015)  *H. capsulatum*: (Chang *et al.* 2017; Garfoot *et al.* 2016) |
| **Natural killer cells**  **NK cells**  (Human primary cells) | Monoculture | Fungal killing | *A. fumigatus:* (Weiss *et al.* 2018)  *C. albicans*: (Li *et al.* 2018)  *C. neoformans:* (Ma *et al.* 2004; Wiseman *et al.* 2007) |
|  |  | Transcriptomics | *C. albicans*: (Hellwig *et al.* 2016) |
|  |  | Chemokines | *A. fumigatus:* (Marischen *et al.* 2018) |
|  |  | Cytokine release | *A. fumigatus:* (Bouzani *et al.* 2011; Santiago *et al.* 2018)  *C. albicans:* (Marolda *et al.* 2020; Voigt *et al.* 2014) |
| **NK cells**  (Human primary cells)  **+ DCs**  (Human primary differentiated cells) | Co-culture | NK-DC cross talk | *A. fumigatus:* (Weiss *et al.* 2018) |
| **DCs**  (Human primary differentiated cells)  **+ T-cells**  (Human primary cells) | Co-culture | Maturation | *A. fumigatus:* (Stephen-Victor *et al.* 2017) |
|  |  | Cytokine release | *C. albicans:* (van der Does *et al.* 2012) |
|  |  | Cytokine release | *A. fumigatus:* (Becker *et al.* 2016; Page *et al.* 2018)  *C. albicans*: (Alvarez-Rueda *et al.* 2020; Estrada-Mata *et al.* 2015; Li *et al.* 2016)  *C. neoformans:* (Mora *et al.* 2017; Siddiqui *et al.* 2006) |
| **Whole blood model**  (Human/Mouse) | Multiculture | Fungal killing | *C. albicans*: (Duggan *et al.* 2015; Hunniger *et al.* 2014) |
|  |  | Transcriptomics | *A. fumigatus:* (Dix *et al.* 2015)  *C. albicans:* (Fradin *et al.* 2005; Kämmer *et al.* 2020) |
|  |  | Cytokine release | *A. fumigatus:* (Oesterreicher *et al.* 2019) |
|  |  | Platelet interaction | *A. fumigatus:* (Fréalle *et al.* 2018)  *C. albicans:* (Eberl *et al.* 2019) |

**References Tab. S1:**

Alsina-Beauchamp D, Escos A, Fajardo P *et al.* Myeloid cell deficiency of p38gamma/p38delta protects against candidiasis and regulates antifungal immunity. *EMBO molecular medicine* 2018;**10**.

Alvarez-Rueda N, Rouges C, Touahri A *et al.* In vitro immune responses of human PBMCs against Candida albicans reveals fungal and leucocyte phenotypes associated with fungal persistence. *Scientific reports* 2020;**10**: 6211.

Alvarez M, Burn T, Luo Y *et al.* The outcome of Cryptococcus neoformans intracellular pathogenesis in human monocytes. *BMC microbiology* 2009;**9**: 51.

Arce Miranda JE, Baronetti JL, Sotomayor CE *et al.* Oxidative and nitrosative stress responses during macrophage-Candida albicans biofilm interaction. *Medical mycology* 2019;**57**: 101-13.

Bain JM, Louw J, Lewis LE *et al.* Candida albicans hypha formation and mannan masking of β-glucan inhibit macrophage phagosome maturation. *mBio* 2014;**5**: e01874.

Baltazar LM, Zamith-Miranda D, Burnet MC *et al.* Concentration-dependent protein loading of extracellular vesicles released by Histoplasma capsulatum after antibody treatment and its modulatory action upon macrophages. *Scientific reports* 2018;**8**: 8065.

Barluzzi R, Brozzetti A, Delfino D *et al.* Role of the capsule in microglial cell-Cryptococcus neoformans interaction: impairment of antifungal activity but not of secretory functions. *Medical mycology* 1998;**36**: 189-97.

Bazan SB, Walch-Rückheim B, Schmitt MJ *et al.* Maturation and cytokine pattern of human dendritic cells in response to different yeasts. *Medical microbiology and immunology* 2018;**207**: 75-81.

Becker KL, Aimanianda V, Wang X *et al.* Aspergillus Cell Wall Chitin Induces Anti- and Proinflammatory Cytokines in Human PBMCs via the Fc-gamma Receptor/Syk/PI3K Pathway. *mBio* 2016;**7**.

Behrens NE, Lipke PN, Pilling D *et al.* Serum Amyloid P Component Binds Fungal Surface Amyloid and Decreases Human Macrophage Phagocytosis and Secretion of Inflammatory Cytokines. *mBio* 2019;**10**.

Bouzani M, Ok M, McCormick A *et al.* Human NK cells display important antifungal activity against Aspergillus fumigatus, which is directly mediated by IFN-γ release. *J Immunol* 2011;**187**: 1369-76.

Boyle KB, Gyori D, Sindrilaru A *et al.* Class IA phosphoinositide 3-kinase β and δ regulate neutrophil oxidase activation in response to Aspergillus fumigatus hyphae. *J Immunol* 2011;**186**: 2978-89.

Brunel SF, Bain JM, King J *et al.* Live Imaging of Antifungal Activity by Human Primary Neutrophils and Monocytes in Response to A. fumigatus. *Journal of visualized experiments : JoVE* 2017, DOI 10.3791/55444.

Brunel SF, Willment JA, Brown GD *et al.* Aspergillus-induced superoxide production by cystic fibrosis phagocytes is associated with disease severity. *ERJ open research* 2018;**4**.

Bruns S, Kniemeyer O, Hasenberg M *et al.* Production of extracellular traps against Aspergillus fumigatus in vitro and in infected lung tissue is dependent on invading neutrophils and influenced by hydrophobin RodA. *PLoS pathogens* 2010;**6**: e1000873.

Bryan AM, Farnoud AM, Mor V *et al.* Macrophage cholesterol depletion and its effect on the phagocytosis of Cryptococcus neoformans. *Journal of visualized experiments : JoVE* 2014, DOI 10.3791/52432.

Camilli G, Eren E, Williams DL *et al.* Impaired phagocytosis directs human monocyte activation in response to fungal derived beta-glucan particles. *European journal of immunology* 2018;**48**: 757-70.

Campos-Garcia L, Jimenez-Valdes RJ, Hernandez-Bello R *et al.* Candida albicans and non-albicans Isolates from Bloodstream Have Different Capacities to Induce Neutrophil Extracellular Traps. *Journal of fungi (Basel, Switzerland)* 2019;**5**.

Chang TH, Huang JH, Lin HC *et al.* Dectin-2 is a primary receptor for NLRP3 inflammasome activation in dendritic cell response to Histoplasma capsulatum. *PLoS pathogens* 2017;**13**: e1006485.

Charlier C, Nielsen K, Daou S *et al.* Evidence of a role for monocytes in dissemination and brain invasion by Cryptococcus neoformans. *Infection and immunity* 2009;**77**: 120-7.

Chew SY, Ho KL, Cheah YK *et al.* Glyoxylate cycle gene ICL1 is essential for the metabolic flexibility and virulence of Candida glabrata. *Scientific reports* 2019;**9**: 2843.

Cho SY, Kwon EY, Choi SM *et al.* Immunomodulatory effect of mesenchymal stem cells on the immune response of macrophages stimulated by Aspergillus fumigatus conidia. *Medical mycology* 2016;**54**: 377-83.

Clark HL, Abbondante S, Minns MS *et al.* Protein Deiminase 4 and CR3 Regulate Aspergillus fumigatus and beta-Glucan-Induced Neutrophil Extracellular Trap Formation, but Hyphal Killing Is Dependent Only on CR3. *Frontiers in immunology* 2018;**9**: 1182.

Coady A, Sil A. MyD88-dependent signaling drives host survival and early cytokine production during Histoplasma capsulatum infection. *Infection and immunity* 2015;**83**: 1265-75.

Coenjaerts FE, Walenkamp AM, Mwinzi PN *et al.* Potent inhibition of neutrophil migration by cryptococcal mannoprotein-4-induced desensitization. *J Immunol* 2001;**167**: 3988-95.

Cottier F, Sherrington S, Cockerill S *et al.* Remasking of Candida albicans beta-Glucan in Response to Environmental pH Is Regulated by Quorum Sensing. *mBio* 2019;**10**.

Dai J, Liang Y, Li H *et al.* Vitamin D enhances resistance to aspergillus fumigatus in mice via inhibition of excessive autophagy. *American journal of translational research* 2018;**10**: 381-91.

Dasari P, Shopova IA, Stroe M *et al.* Aspf2 From Aspergillus fumigatus Recruits Human Immune Regulators for Immune Evasion and Cell Damage. *Frontiers in immunology* 2018;**9**: 1635.

de Albuquerque JAT, Banerjee PP, Castoldi A *et al.* The Role of AIRE in the Immunity Against Candida Albicans in a Model of Human Macrophages. *Frontiers in immunology* 2018;**9**: 567.

Deepe GS, Jr., Buesing WR. Deciphering the pathways of death of Histoplasma capsulatum-infected macrophages: implications for the immunopathogenesis of early infection. *Journal of immunology (Baltimore, Md : 1950)* 2012;**188**: 334-44.

Dix A, Hunniger K, Weber M *et al.* Biomarker-based classification of bacterial and fungal whole-blood infections in a genome-wide expression study. *Frontiers in microbiology* 2015;**6**: 171.

Dominguez-Andres J, Arts RJW, Ter Horst R *et al.* Rewiring monocyte glucose metabolism via C-type lectin signaling protects against disseminated candidiasis. *PLoS pathogens* 2017;**13**: e1006632.

Drummond RA, Collar AL, Swamydas M *et al.* CARD9-Dependent Neutrophil Recruitment Protects against Fungal Invasion of the Central Nervous System. *PLoS pathogens* 2015;**11**: e1005293.

Drummond RA, Swamydas M, Oikonomou V *et al.* CARD9(+) microglia promote antifungal immunity via IL-1β- and CXCL1-mediated neutrophil recruitment. *Nat Immunol* 2019;**20**: 559-70.

Duggan S, Essig F, Hunniger K *et al.* Neutrophil activation by Candida glabrata but not Candida albicans promotes fungal uptake by monocytes. *Cellular microbiology* 2015;**17**: 1259-76.

Duvenage L, Walker LA, Bojarczuk A *et al.* Inhibition of Classical and Alternative Modes of Respiration in Candida albicans Leads to Cell Wall Remodeling and Increased Macrophage Recognition. *mBio* 2019;**10**.

Eberl C, Speth C, Jacobsen ID *et al.* Candida: Platelet Interaction and Platelet Activity in vitro. *J Innate Immun* 2019;**11**: 52-62.

Ellett F, Jorgensen J, Frydman GH *et al.* Neutrophil Interactions Stimulate Evasive Hyphal Branching by Aspergillus fumigatus. *PLoS pathogens* 2017;**13**: e1006154.

Essig F, Hunniger K, Dietrich S *et al.* Human neutrophils dump Candida glabrata after intracellular killing. *Fungal genetics and biology : FG & B* 2015;**84**: 37-40.

Estrada-Mata E, Navarro-Arias MJ, Pérez-García LA *et al.* Members of the Candida parapsilosis Complex and Candida albicans are Differentially Recognized by Human Peripheral Blood Mononuclear Cells. *Frontiers in microbiology* 2015;**6**: 1527.

Fliesser M, Wallstein M, Kurzai O *et al.* Hypoxia attenuates anti-Aspergillus fumigatus immune responses initiated by human dendritic cells. *Mycoses* 2016;**59**: 503-8.

Fradin C, De Groot P, MacCallum D *et al.* Granulocytes govern the transcriptional response, morphology and proliferation of Candida albicans in human blood. *Molecular microbiology* 2005;**56**: 397-415.

Fréalle E, Gosset P, Leroy S *et al.* In vitro coagulation triggers anti-Aspergillus fumigatus neutrophil response. *Future microbiology* 2018;**13**: 659-69.

Friedrich D, Zapf D, Lohse B *et al.* The HIF-1alpha/LC3-II Axis Impacts Fungal Immunity in Human Macrophages. *Infection and immunity* 2019;**87**.

Garfoot AL, Shen Q, Wuthrich M *et al.* The Eng1 beta-Glucanase Enhances Histoplasma Virulence by Reducing beta-Glucan Exposure. *mBio* 2016;**7**: e01388-15.

Gazendam RP, van Hamme JL, Tool AT *et al.* Human Neutrophils Use Different Mechanisms To Kill Aspergillus fumigatus Conidia and Hyphae: Evidence from Phagocyte Defects. *Journal of immunology (Baltimore, Md : 1950)* 2016;**196**: 1272-83.

Gildea LA, Morris RE, Newman SL. Histoplasma capsulatum yeasts are phagocytosed via very late antigen-5, killed, and processed for antigen presentation by human dendritic cells. *Journal of immunology (Baltimore, Md : 1950)* 2001;**166**: 1049-56.

Gonçalves SM, Duarte-Oliveira C, Campos CF *et al.* Phagosomal removal of fungal melanin reprograms macrophage metabolism to promote antifungal immunity. *Nature communications* 2020;**11**: 2282.

Gresnigt MS, Becker KL, Leenders F *et al.* Differential Kinetics of Aspergillus nidulans and Aspergillus fumigatus Phagocytosis. *J Innate Immun* 2018a;**10**: 145-60.

Gresnigt MS, Cunha C, Jaeger M *et al.* Genetic deficiency of NOD2 confers resistance to invasive aspergillosis. *Nature communications* 2018b;**9**: 2636.

Grimm MJ, D'Auria AC, Segal BH. Assessing anti-fungal activity of isolated alveolar macrophages by confocal microscopy. *Journal of visualized experiments : JoVE* 2014, DOI 10.3791/51678.

Grondman I, Arts RJW, Koch RM *et al.* Frontline Science: Endotoxin-induced immunotolerance is associated with loss of monocyte metabolic plasticity and reduction of oxidative burst. *Journal of leukocyte biology* 2019;**106**: 11-25.

Guiducci E, Lemberg C, Kung N *et al.* Candida albicans-Induced NETosis Is Independent of Peptidylarginine Deiminase 4. *Frontiers in immunology* 2018;**9**: 1573.

Guimaraes AJ, de Cerqueira MD, Zamith-Miranda D *et al.* Host membrane glycosphingolipids and lipid microdomains facilitate Histoplasma capsulatum internalisation by macrophages. *Cellular microbiology* 2019;**21**: e12976.

Haider M, Dambuza IM, Asamaphan P *et al.* The pattern recognition receptors dectin-2, mincle, and FcRγ impact the dynamics of phagocytosis of Candida, Saccharomyces, Malassezia, and Mucor species. *PloS one* 2019;**14**: e0220867.

Halder LD, Abdelfatah MA, Jo EA *et al.* Factor H Binds to Extracellular DNA Traps Released from Human Blood Monocytes in Response to Candida albicans. *Frontiers in immunology* 2016;**7**: 671.

Hansakon A, Mutthakalin P, Ngamskulrungroj P *et al.* Cryptococcus neoformans and Cryptococcus gattii clinical isolates from Thailand display diverse phenotypic interactions with macrophages. *Virulence* 2019;**10**: 26-36.

Hefter M, Lother J, Weiss E *et al.* Human primary myeloid dendritic cells interact with the opportunistic fungal pathogen Aspergillus fumigatus via the C-type lectin receptor Dectin-1. *Medical mycology* 2017;**55**: 573-8.

Hellwig D, Voigt J, Bouzani M *et al.* Candida albicans Induces Metabolic Reprogramming in Human NK Cells and Responds to Perforin with a Zinc Depletion Response. *Frontiers in microbiology* 2016;**7**: 750.

Hopke A, Scherer A, Kreuzburg S *et al.* Neutrophil swarming delays the growth of clusters of pathogenic fungi. *Nature communications* 2020;**11**: 2031.

Hunniger K, Lehnert T, Bieber K *et al.* A virtual infection model quantifies innate effector mechanisms and Candida albicans immune escape in human blood. *PLoS computational biology* 2014;**10**: e1003479.

Ifrim DC, Quintin J, Courjol F *et al.* The Role of Dectin-2 for Host Defense Against Disseminated Candidiasis. *Journal of interferon & cytokine research : the official journal of the International Society for Interferon and Cytokine Research* 2016;**36**: 267-76.

Jiang HH, Zhang YJ, Sun YZ *et al.* Cell wall mannoprotein of Candida albicans polarizes macrophages and affects proliferation and apoptosis through activation of the Akt signal pathway. *International immunopharmacology* 2019;**72**: 308-21.

Johnson CJ, Kernien JF, Hoyer AR *et al.* Mechanisms involved in the triggering of neutrophil extracellular traps (NETs) by Candida glabrata during planktonic and biofilm growth. *Scientific reports* 2017;**7**: 13065.

Jones CN, Ellett F, Robertson AL *et al.* Bifunctional Small Molecules Enhance Neutrophil Activities Against Aspergillus fumigatus in vivo and in vitro. *Frontiers in immunology* 2019;**10**: 644.

Kämmer P, McNamara S, Wolf T *et al.* Survival Strategies of Pathogenic Candida Species in Human Blood Show Independent and Specific Adaptations. *mBio* 2020;**11**.

Kasper L, Konig A, Koenig PA *et al.* The fungal peptide toxin Candidalysin activates the NLRP3 inflammasome and causes cytolysis in mononuclear phagocytes. *Nature communications* 2018;**9**: 4260.

Kaya EG, Ozbilge H, Ustundag MB *et al.* The effects on immune response of levamisole treatment following infection of U-937 macrophages with Candida albicans. *Acta Microbiol Immunol Hung* 2011;**58**: 279-88.

Kelly RM, Chen J, Yauch LE *et al.* Opsonic requirements for dendritic cell-mediated responses to Cryptococcus neoformans. *Infection and immunity* 2005;**73**: 592-8.

Kuhn DM, Vyas VK. The Candida glabrata adhesin Epa1p causes adhesion, phagocytosis, and cytokine secretion by innate immune cells. *FEMS yeast research* 2012;**12**: 398-414.

Kyrmizi I, Ferreira H, Carvalho A *et al.* Calcium sequestration by fungal melanin inhibits calcium-calmodulin signalling to prevent LC3-associated phagocytosis. *Nature microbiology* 2018;**3**: 791-803.

Leonhardt J, Grosse S, Marx C *et al.* Candida albicans beta-Glucan Differentiates Human Monocytes Into a Specific Subset of Macrophages. *Frontiers in immunology* 2018;**9**: 2818.

Li SS, Ogbomo H, Mansour MK *et al.* Identification of the fungal ligand triggering cytotoxic PRR-mediated NK cell killing of Cryptococcus and Candida. *Nature communications* 2018;**9**: 751.

Li Y, Oosting M, Deelen P *et al.* Inter-individual variability and genetic influences on cytokine responses to bacteria and fungi. *Nat Med* 2016;**22**: 952-60.

Liu NN, Uppuluri P, Broggi A *et al.* Intersection of phosphate transport, oxidative stress and TOR signalling in Candida albicans virulence. *PLoS pathogens* 2018;**14**: e1007076.

Liu Y, Ou Y, Sun L *et al.* Alcohol dehydrogenase of Candida albicans triggers differentiation of THP-1 cells into macrophages. *Journal of advanced research* 2019;**18**: 137-45.

Lopez CM, Wallich R, Riesbeck K *et al.* Candida albicans uses the surface protein Gpm1 to attach to human endothelial cells and to keratinocytes via the adhesive protein vitronectin. *PloS one* 2014;**9**: e90796.

Lother J, Breitschopf T, Krappmann S *et al.* Human dendritic cell subsets display distinct interactions with the pathogenic mould Aspergillus fumigatus. *International journal of medical microbiology : IJMM* 2014;**304**: 1160-8.

Loureiro A, Pais C, Sampaio P. Relevance of Macrophage Extracellular Traps in C. albicans Killing. *Frontiers in immunology* 2019;**10**: 2767.

Ma LL, Wang CL, Neely GG *et al.* NK cells use perforin rather than granulysin for anticryptococcal activity. *Journal of immunology (Baltimore, Md : 1950)* 2004;**173**: 3357-65.

Maher CO, Dunne K, Comerford R *et al.* Candida albicans stimulates IL-23 release by human dendritic cells and downstream IL-17 secretion by Vdelta1 T cells. *Journal of immunology (Baltimore, Md : 1950)* 2015;**194**: 5953-60.

Marischen L, Englert A, Schmitt AL *et al.* Human NK cells adapt their immune response towards increasing multiplicities of infection of Aspergillus fumigatus. *BMC immunology* 2018;**19**: 39.

Marolda A, Hünniger K, Böttcher S *et al.* Candida species-dependent release of IL-12 by dendritic cells induces different levels of NK cell stimulation. *The Journal of infectious diseases* 2020, DOI 10.1093/infdis/jiaa035.

Mattern DJ, Schoeler H, Weber J *et al.* Identification of the antiphagocytic trypacidin gene cluster in the human-pathogenic fungus Aspergillus fumigatus. *Applied microbiology and biotechnology* 2015;**99**: 10151-61.

Miramón P, Dunker C, Windecker H *et al.* Cellular responses of Candida albicans to phagocytosis and the extracellular activities of neutrophils are critical to counteract carbohydrate starvation, oxidative and nitrosative stress. *PloS one* 2012;**7**: e52850.

Mora DJ, Ferreira-Paim K, Andrade-Silva LE *et al.* Cytokine patterns in a prospective cohort of HIV-infected patients with cryptococcal meningitis following initiation of antifungal and antiretroviral therapy. *PloS one* 2017;**12**: e0176304.

Munawara U, Small AG, Quach A *et al.* Cytokines regulate complement receptor immunoglobulin expression and phagocytosis of Candida albicans in human macrophages: A control point in anti-microbial immunity. *Scientific reports* 2017;**7**: 4050.

Muñoz JF, Delorey T, Ford CB *et al.* Coordinated host-pathogen transcriptional dynamics revealed using sorted subpopulations and single macrophages infected with Candida albicans. *Nature communications* 2019;**10**: 1607.

Negoro PE, Xu S, Dagher Z *et al.* Spleen Tyrosine Kinase Is a Critical Regulator of Neutrophil Responses to Candida Species. *mBio* 2020;**11**.

Nguyen TNY, Matangkasombut O, Ritprajak P. Differential dendritic cell responses to cell wall mannan of Candida albicans, Candida parapsilosis, and Candida dubliniensis. *Journal of oral science* 2018;**60**: 557-66.

Niemiec MJ, Grumaz C, Ermert D *et al.* Dual transcriptome of the immediate neutrophil and Candida albicans interplay. *BMC genomics* 2017;**18**: 696.

O'Meara TR, Duah K, Guo CX *et al.* High-Throughput Screening Identifies Genes Required for Candida albicans Induction of Macrophage Pyroptosis. *mBio* 2018;**9**.

Oesterreicher Z, Eberl S, Zeitlinger M. Impact of different antimycotics on cytokine levels in an in vitro aspergillosis model in human whole blood. *Infection* 2019, DOI 10.1007/s15010-019-01346-x.

Okai B, Lyall N, Gow NA *et al.* Rab14 regulates maturation of macrophage phagosomes containing the fungal pathogen Candida albicans and outcome of the host-pathogen interaction. *Infection and immunity* 2015;**83**: 1523-35.

Oya E, Solhaug A, Bolling AK *et al.* Pro-inflammatory responses induced by A. fumigatus and A. versicolor in various human macrophage models. *Journal of toxicology and environmental health Part A* 2019;**82**: 483-501.

Page L, Weis P, Muller T *et al.* Evaluation of Aspergillus and Mucorales specific T-cells and peripheral blood mononuclear cell cytokine signatures as biomarkers of environmental mold exposure. *International journal of medical microbiology : IJMM* 2018;**308**: 1018-26.

Pereira PAT, Assis PA, Prado MKB *et al.* Prostaglandins D2 and E2 have opposite effects on alveolar macrophages infected with Histoplasma capsulatum. *Journal of lipid research* 2018;**59**: 195-206.

Pietrella D, Corbucci C, Perito S *et al.* Mannoproteins from Cryptococcus neoformans promote dendritic cell maturation and activation. *Infection and immunity* 2005;**73**: 820-7.

Rieber N, Gazendam RP, Freeman AF *et al.* Extrapulmonary Aspergillus infection in patients with CARD9 deficiency. *JCI Insight* 2016;**1**: e89890.

Rocha JD, Nascimento MT, Decote-Ricardo D *et al.* Capsular polysaccharides from Cryptococcus neoformans modulate production of neutrophil extracellular traps (NETs) by human neutrophils. *Scientific reports* 2015;**5**: 8008.

Röhm M, Grimm MJ, D'Auria AC *et al.* NADPH oxidase promotes neutrophil extracellular trap formation in pulmonary aspergillosis. *Infection and immunity* 2014;**82**: 1766-77.

Roudbary M, Roudbar Mohammadi S, Bozorgmehr M *et al.* The effects of Candida albicans cell wall protein fraction on dendritic cell maturation. *Iran J Immunol* 2009;**6**: 67-74.

Rubino I, Coste A, Le Roy D *et al.* Species-specific recognition of Aspergillus fumigatus by Toll-like receptor 1 and Toll-like receptor 6. *The Journal of infectious diseases* 2012;**205**: 944-54.

Rudkin FM, Raziunaite I, Workman H *et al.* Single human B cell-derived monoclonal anti-Candida antibodies enhance phagocytosis and protect against disseminated candidiasis. *Nature communications* 2018;**9**: 5288.

Salvatori O, Pathirana RU, Kay JG *et al.* Candida albicans Ras1 Inactivation Increases Resistance to Phagosomal Killing by Human Neutrophils. *Infection and immunity* 2018;**86**.

Santiago V, Rezvani K, Sekine T *et al.* Human NK Cells Develop an Exhaustion Phenotype During Polar Degranulation at the Aspergillus fumigatus Hyphal Synapse. *Frontiers in immunology* 2018;**9**: 2344.

Schmidt H, Vlaic S, Kruger T *et al.* Proteomics of Aspergillus fumigatus Conidia-containing Phagolysosomes Identifies Processes Governing Immune Evasion. *Molecular & cellular proteomics : MCP* 2018;**17**: 1084-96.

Scott AJ, Woods JP. Monitoring internalization of Histoplasma capsulatum by mammalian cell lines using a fluorometric microplate assay. *Medical mycology* 2000;**38**: 15-22.

Shen Q, Beucler MJ, Ray SC *et al.* Macrophage activation by IFN-gamma triggers restriction of phagosomal copper from intracellular pathogens. *PLoS pathogens* 2018;**14**: e1007444.

Shimamura S, Miyazaki T, Tashiro M *et al.* Autophagy-Inducing Factor Atg1 Is Required for Virulence in the Pathogenic Fungus Candida glabrata. *Frontiers in microbiology* 2019;**10**: 27.

Siddiqui AA, Shattock RJ, Harrison TS. Role of capsule and interleukin-6 in long-term immune control of Cryptococcus neoformans infection by specifically activated human peripheral blood mononuclear cells. *Infection and immunity* 2006;**74**: 5302-10.

Silva JC, Rodrigues NC, Thompson-Souza GA *et al.* Mac-1 triggers neutrophil DNA extracellular trap formation to Aspergillus fumigatus independently of PAD4 histone citrullination. *Journal of leukocyte biology* 2019, DOI 10.1002/jlb.4a0119-009rr.

Smith LM, Dixon EF, May RC. The fungal pathogen Cryptococcus neoformans manipulates macrophage phagosome maturation. *Cellular microbiology* 2015;**17**: 702-13.

Srivastava M, Bencurova E, Gupta SK *et al.* Aspergillus fumigatus Challenged by Human Dendritic Cells: Metabolic and Regulatory Pathway Responses Testify a Tight Battle. *Frontiers in cellular and infection microbiology* 2019;**9**: 168.

Stephen-Victor E, Karnam A, Fontaine T *et al.* Aspergillus fumigatus Cell Wall α-(1,3)-Glucan Stimulates Regulatory T-Cell Polarization by Inducing PD-L1 Expression on Human Dendritic Cells. *The Journal of infectious diseases* 2017;**216**: 1281-94.

Stukes S, Casadevall A. Visualizing non-lytic exocytosis of Cryptococcus neoformans from macrophages using digital light microscopy. *Journal of visualized experiments : JoVE* 2014, DOI 10.3791/52084: e52084.

Sun D, Zhang M, Liu G *et al.* Real-Time Imaging of Interactions of Neutrophils with Cryptococcus neoformans Demonstrates a Crucial Role of Complement C5a-C5aR Signaling. *Infection and immunity* 2016;**84**: 216-29.

Sun H, Xu XY, Tian XL *et al.* Activation of NF-κB and respiratory burst following Aspergillus fumigatus stimulation of macrophages. *Immunobiology* 2014;**219**: 25-36.

Tagliari L, Toledo MS, Lacerda TG *et al.* Membrane microdomain components of Histoplasma capsulatum yeast forms, and their role in alveolar macrophage infectivity. *Biochimica et biophysica acta* 2012;**1818**: 458-66.

Thompson-Souza GA, Santos GMP, Silva JC *et al.* Histoplasma capsulatum-induced extracellular DNA trap release in human neutrophils. *Cellular microbiology* 2020, DOI 10.1111/cmi.13195: e13195.

Thompson A, Griffiths JS, Walker L *et al.* Dependence on Dectin-1 Varies With Multiple Candida Species. *Frontiers in microbiology* 2019;**10**: 1800.

Urban CF, Reichard U, Brinkmann V *et al.* Neutrophil extracellular traps capture and kill Candida albicans yeast and hyphal forms. *Cellular microbiology* 2006;**8**: 668-76.

van der Does AM, Joosten SA, Vroomans E *et al.* The antimicrobial peptide hLF1-11 drives monocyte-dendritic cell differentiation toward dendritic cells that promote antifungal responses and enhance Th17 polarization. *J Innate Immun* 2012;**4**: 284-92.

Van Prooyen N, Henderson CA, Hocking Murray D *et al.* CD103+ Conventional Dendritic Cells Are Critical for TLR7/9-Dependent Host Defense against Histoplasma capsulatum, an Endemic Fungal Pathogen of Humans. *PLoS pathogens* 2016;**12**: e1005749.

Vaz C, Reales-Calderon JA, Pitarch A *et al.* Enrichment of ATP Binding Proteins Unveils Proteomic Alterations in Human Macrophage Cell Death, Inflammatory Response, and Protein Synthesis after Interaction with Candida albicans. *Journal of proteome research* 2019;**18**: 2139-59.

Veloso Júnior PHH, Simon KS, de Castro RJA *et al.* Peptides ToAP3 and ToAP4 decrease release of inflammatory cytokines through TLR-4 blocking. *Biomed Pharmacother* 2019;**118**: 109152.

Vivas W, Leonhardt I, Hünniger K *et al.* Multiple Signaling Pathways Involved in Human Dendritic Cell Maturation Are Affected by the Fungal Quorum-Sensing Molecule Farnesol. *J Immunol* 2019;**203**: 2959-69.

Voigt J, Hunniger K, Bouzani M *et al.* Human natural killer cells acting as phagocytes against Candida albicans and mounting an inflammatory response that modulates neutrophil antifungal activity. *The Journal of infectious diseases* 2014;**209**: 616-26.

Walsh NM, Wuthrich M, Wang H *et al.* Characterization of C-type lectins reveals an unexpectedly limited interaction between Cryptococcus neoformans spores and Dectin-1. *PloS one* 2017;**12**: e0173866.

Wang W, Deng Z, Wu H *et al.* A small secreted protein triggers a TLR2/4-dependent inflammatory response during invasive Candida albicans infection. *Nature communications* 2019;**10**: 1015.

Weiss E, Ziegler S, Fliesser M *et al.* First Insights in NK-DC Cross-Talk and the Importance of Soluble Factors During Infection With Aspergillus fumigatus. *Frontiers in cellular and infection microbiology* 2018;**8**: 288.

Wellington M, Dolan K, Krysan DJ. Live Candida albicans suppresses production of reactive oxygen species in phagocytes. *Infection and immunity* 2009;**77**: 405-13.

Wiseman JC, Ma LL, Marr KJ *et al.* Perforin-dependent cryptococcal microbicidal activity in NK cells requires PI3K-dependent ERK1/2 signaling. *Journal of immunology (Baltimore, Md : 1950)* 2007;**178**: 6456-64.

Wolf JE, Kerchberger V, Kobayashi GS *et al.* Modulation of the macrophage oxidative burst by Histoplasma capsulatum. *J Immunol* 1987;**138**: 582-6.

Wu SY, Weng CL, Jheng MJ *et al.* Candida albicans triggers NADPH oxidase-independent neutrophil extracellular traps through dectin-2. *PLoS pathogens* 2019a;**15**: e1008096.

Wu Y, Du S, Johnson JL *et al.* Microglia and amyloid precursor protein coordinate control of transient Candida cerebritis with memory deficits. *Nature communications* 2019b;**10**: 58.

Wu Y, Xu H, Li L *et al.* Susceptibility to Aspergillus Infections in Rats with Chronic Obstructive Pulmonary Disease via Deficiency Function of Alveolar Macrophages and Impaired Activation of TLR2. *Inflammation* 2016;**39**: 1310-8.

Youseff BH, Holbrook ED, Smolnycki KA *et al.* Extracellular superoxide dismutase protects Histoplasma yeast cells from host-derived oxidative stress. *PLoS pathogens* 2012;**8**: e1002713.

Zawrotniak M, Wojtalik K, Rapala-Kozik M. Farnesol, a Quorum-Sensing Molecule of Candida Albicans Triggers the Release of Neutrophil Extracellular Traps. *Cells* 2019;**8**.

Zhang P, Xin X, Fang L *et al.* HMGB1 mediates Aspergillus fumigatus-induced inflammatory response in alveolar macrophages of COPD mice via activating MyD88/NF-kappaB and syk/PI3K signalings. *International immunopharmacology* 2017;**53**: 125-32.

**Table S2: Overview about the different lung *in vitro* models, their structure, possible read outs and applications for the different fungi**

| **Model** | **Structure** | **Readout** | **Reference** |
| --- | --- | --- | --- |
| **Alveolar epithelial cells**  **A549**  (Human cell line) | Monoculture | Adhesion | *A. fumigatus*: (Gravelat *et al.* 2010; Xu *et al.* 2012)  *C. neoformans*: (Choo *et al.* 2015; Ganendren *et al.* 2006; Teixeira *et al.* 2014)  *H. capsulatum*: (Pitangui *et al.* 2012) |
|  |  | Endocytosis | *A. fumigatus:* (Amin *et al.* 2014; Liu *et al.* 2016; Xu *et al.* 2012) |
|  |  | Damage | *A. fumigatus:* (Bertuzzi *et al.* 2014; Dasari *et al.* 2019; Ejzykowicz *et al.* 2010) |
|  |  | Cell Detachment | *A. fumigatus:* (Bertuzzi *et al.* 2014; Kogan *et al.* 2004) |
|  |  | Transcriptomics | *A. fumigatus:* (Jepsen *et al.* 2018; Takahashi-Nakaguchi *et al.* 2018; Watkins *et al.* 2018) |
|  |  | Proteomics | *A. fumigatus:* (Margalit *et al.* 2020; Voltersen *et al.* 2018) |
|  |  | Metabolomics | *C. neoformans*: (Liew *et al.* 2016) |
|  |  | Cytokine release | *C. neoformans:* (Barbosa *et al.* 2007)  *H. capsulatum*: (Alcantara *et al.* 2020; Maza and Suzuki 2016) |
| **Human bronchial epithelial or small airway epithelial**  **Cells**  **HBE, SAE**  (Human primary cells) | Monoculture | Cytokine release | *A. fumigatus*: (Sun *et al.* 2012)  *C. neoformans:* (Guillot *et al.* 2008) |
|  | Monoculture differentiated in an ALI | Transcriptomics  Proteomics | *A. fumigatus*: (Toor *et al.* 2018) |
| **A549**  (Human cell line)  **+ DCs**  (Human primary differentiated cells) | Co-culture on Transwell  (top: A549 + DCs) | Damage  Membrane integrity  Cytokine release | *A. fumigatus*: (Morton *et al.* 2018) |
| **Human pulmonary artery endothelial cells HPAECs**  (Human cell line)  **+ A549**  (Human cell line) | Co-culture  on  Transwell  (top: A549  bottom: HPAECs) | Microscopy  Kinetics | *A. fumigatus*: (Hope *et al.* 2007) |
|  |  | Cytokine release | *A. fumigatus*: (Belic *et al.* 2018) |
| **HBE, SAE**  (Human primary cells)  **+ DCs**  (Human primary differentiated cells)  **+ Macrophages**  (Human primary cells) | Co-culture  differentiated in an ALI | Cytokine release | *A. fumigatus*: (Chandorkar *et al.* 2017) |
| **HPAECs**  (Human cell line)  **+ A549**  (Human cell line)  **+ monocyte-derived DCs and myeloid DCs**  (Human primary differentiated cells) | Co-culture  on  Transwell  (top: A549 + DCs  bottom: HPAECs) | Transcriptomics | *A. fumigatus*: (Morton *et al.* 2014) |
| **Lung-on-chip model**  alveolar epithelium  **+**  microvascular endothelium | Bilayer with ALI on one side and flow on the other side | Model establishment | (Deinhardt-Emmer et al. 2020) |

**References Tab. S2:**

Alcantara C, Almeida BR, Barros B *et al.* Histoplasma capsulatum chemotypes I and II induce IL-8 secretion in lung epithelial cells in distinct manners. *Medical mycology* 2020, DOI 10.1093/mmy/myaa006.

Amin S, Thywissen A, Heinekamp T *et al.* Melanin dependent survival of Apergillus fumigatus conidia in lung epithelial cells. *International journal of medical microbiology : IJMM* 2014;**304**: 626-36.

Barbosa FM, Fonseca FL, Figueiredo RT *et al.* Binding of glucuronoxylomannan to the CD14 receptor in human A549 alveolar cells induces interleukin-8 production. *Clinical and vaccine immunology : CVI* 2007;**14**: 94-8.

Belic S, Page L, Lazariotou M *et al.* Comparative Analysis of Inflammatory Cytokine Release and Alveolar Epithelial Barrier Invasion in a Transwell((R)) Bilayer Model of Mucormycosis. *Frontiers in microbiology* 2018;**9**: 3204.

Bertuzzi M, Schrettl M, Alcazar-Fuoli L *et al.* The pH-responsive PacC transcription factor of Aspergillus fumigatus governs epithelial entry and tissue invasion during pulmonary aspergillosis. *PLoS pathogens* 2014;**10**: e1004413.

Chandorkar P, Posch W, Zaderer V *et al.* Fast-track development of an in vitro 3D lung/immune cell model to study Aspergillus infections. *Scientific reports* 2017;**7**: 11644.

Choo KK, Chong PP, Ho AS *et al.* The role of host microfilaments and microtubules during opsonin-independent interactions of Cryptococcus neoformans with mammalian lung cells. *European journal of clinical microbiology & infectious diseases : official publication of the European Society of Clinical Microbiology* 2015;**34**: 2421-7.

Dasari P, Koleci N, Shopova IA *et al.* Enolase From Aspergillus fumigatus Is a Moonlighting Protein That Binds the Human Plasma Complement Proteins Factor H, FHL-1, C4BP, and Plasminogen. *Frontiers in immunology* 2019;**10**: 2573.

Deinhardt-Emmer S, Rennert K, Schicke E *et al.* Co-infection with Staphylococcus aureus after primary influenza virus infection leads to damage of the endothelium in a human alveolus-on-a-chip model. *Biofabrication* 2020;**12**: 025012.

Ejzykowicz DE, Solis NV, Gravelat FN *et al.* Role of Aspergillus fumigatus DvrA in host cell interactions and virulence. *Eukaryotic cell* 2010;**9**: 1432-40.

Ganendren R, Carter E, Sorrell T *et al.* Phospholipase B activity enhances adhesion of Cryptococcus neoformans to a human lung epithelial cell line. *Microbes and infection* 2006;**8**: 1006-15.

Gravelat FN, Ejzykowicz DE, Chiang LY *et al.* Aspergillus fumigatus MedA governs adherence, host cell interactions and virulence. *Cellular microbiology* 2010;**12**: 473-88.

Guillot L, Carroll SF, Badawy M *et al.* Cryptococcus neoformans induces IL-8 secretion and CXCL1 expression by human bronchial epithelial cells. *Respir Res* 2008;**9**: 9.

Hope WW, Kruhlak MJ, Lyman CA *et al.* Pathogenesis of Aspergillus fumigatus and the kinetics of galactomannan in an in vitro model of early invasive pulmonary aspergillosis: implications for antifungal therapy. *The Journal of infectious diseases* 2007;**195**: 455-66.

Jepsen CS, Dubey LK, Colmorten KB *et al.* FIBCD1 Binds Aspergillus fumigatus and Regulates Lung Epithelial Response to Cell Wall Components. *Frontiers in immunology* 2018;**9**: 1967.

Kogan TV, Jadoun J, Mittelman L *et al.* Involvement of secreted Aspergillus fumigatus proteases in disruption of the actin fiber cytoskeleton and loss of focal adhesion sites in infected A549 lung pneumocytes. *The Journal of infectious diseases* 2004;**189**: 1965-73.

Liew KL, Jee JM, Yap I *et al.* In Vitro Analysis of Metabolites Secreted during Infection of Lung Epithelial Cells by Cryptococcus neoformans. *PloS one* 2016;**11**: e0153356.

Liu H, Lee MJ, Solis NV *et al.* Aspergillus fumigatus CalA binds to integrin alpha5beta1 and mediates host cell invasion. *Nature microbiology* 2016;**2**: 16211.

Margalit A, Kavanagh K, Carolan JC. Characterization of the Proteomic Response of A549 Cells Following Sequential Exposure to Aspergillus fumigatus and Pseudomonas aeruginosa. *Journal of proteome research* 2020;**19**: 279-91.

Maza PK, Suzuki E. Histoplasma capsulatum-Induced Cytokine Secretion in Lung Epithelial Cells Is Dependent on Host Integrins, Src-Family Kinase Activation, and Membrane Raft Recruitment. *Frontiers in microbiology* 2016;**7**: 580.

Morton CO, Fliesser M, Dittrich M *et al.* Gene expression profiles of human dendritic cells interacting with Aspergillus fumigatus in a bilayer model of the alveolar epithelium/endothelium interface. *PloS one* 2014;**9**: e98279.

Morton CO, Wurster S, Fliesser M *et al.* Validation of a simplified in vitro Transwell((R)) model of the alveolar surface to assess host immunity induced by different morphotypes of Aspergillus fumigatus. *International journal of medical microbiology : IJMM* 2018;**308**: 1009-17.

Pitangui NS, Sardi JC, Silva JF *et al.* Adhesion of Histoplasma capsulatum to pneumocytes and biofilm formation on an abiotic surface. *Biofouling* 2012;**28**: 711-8.

Sun WK, Lu X, Li X *et al.* Dectin-1 is inducible and plays a crucial role in Aspergillus-induced innate immune responses in human bronchial epithelial cells. *European journal of clinical microbiology & infectious diseases : official publication of the European Society of Clinical Microbiology* 2012;**31**: 2755-64.

Takahashi-Nakaguchi A, Sakai K, Takahashi H *et al.* Aspergillus fumigatus adhesion factors in dormant conidia revealed through comparative phenotypic and transcriptomic analyses. *Cellular microbiology* 2018;**20**.

Teixeira PA, Penha LL, Mendonca-Previato L *et al.* Mannoprotein MP84 mediates the adhesion of Cryptococcus neoformans to epithelial lung cells. *Frontiers in cellular and infection microbiology* 2014;**4**: 106.

Toor A, Culibrk L, Singhera GK *et al.* Transcriptomic and proteomic host response to Aspergillus fumigatus conidia in an air-liquid interface model of human bronchial epithelium. *PloS one* 2018;**13**: e0209652.

Voltersen V, Blango MG, Herrmann S *et al.* Proteome Analysis Reveals the Conidial Surface Protein CcpA Essential for Virulence of the Pathogenic Fungus Aspergillus fumigatus. *mBio* 2018;**9**.

Watkins TN, Liu H, Chung M *et al.* Comparative transcriptomics of Aspergillus fumigatus strains upon exposure to human airway epithelial cells. *Microbial genomics* 2018;**4**.

Xu XY, Shi Y, Zhang PP *et al.* E-cadherin mediates adhesion and endocytosis of Aspergillus fumigatus blastospores in human epithelial cells. *Chinese medical journal* 2012;**125**: 617-21.

**Table S3: Overview about the different *in vitro* models for the gastrointestinal tract and vaginal tract, their structure, possible read outs and applications for *Candida* spp.**

| **Model** | **Structure** | **Readout** | **Reference** |
| --- | --- | --- | --- |
| 1. **Oral cavity** | | | |
| **Keratinocyte cells**  **TR146**  (Human cell line) | Monoculture | Invasion | *C. albicans:* (Puri *et al.* 2019) |
|  |  | Damage | *C. albicans:* (Wilson *et al.* 2014) |
|  |  | Transcriptomics | *C. albicans:* (McCall *et al.* 2018; Schaller *et al.* 1998) |
|  |  | Cytokine release | *C. albicans:* (Ho *et al.* 2019; Verma *et al.* 2017; Verma *et al.* 2018) |
| **Tongue cells**  **SCC15**  (Human cell line) | Monoculture | Invasion | *C. albicans:* (Villar et al. 2007) |
|  |  | Damage | *C. albicans:* (Kumar et al. 2015) |
|  |  | Cytokine release | *C. albicans:* (Dongari-Bagtzoglou and Kashleva 2003) |
| **Immortalized oral mucosal cells** **OKF6/TERT-2**  (Human cell line) | Monoculture | Invasion | *C. albicans:* (Solis et al. 2017; Swidergall et al. 2018; Zhu et al. 2012) |
|  |  | Damage | *C. albicans:* (Liu et al. 2014; Solis et al. 2018) |
|  |  | Transcriptomics | *C. albicans:* (Liu *et al.* 2015) |
|  |  | Live cell imaging | *C. albicans:* (Wollert *et al.* 2012) |
| **Human palate epithelial cells**  **HPECs**  (Human primary cells) | Monoculture | Apoptosis | *C. albicans:* (Casaroto *et al.* 2019) |
|  |  | Host gene expression | *C. albicans:* (Offenbacher *et al.* 2019) |
| **TR146**  (Human cell line)  **+ Fibroblasts**  (Human primary cells) | Co-culture | Adhesion  Gene expression | *C. albicans:* (Morse *et al.* 2018) |
| **Reconstituted human oral epithelium**  **RHOE**  (cell line based) | 3D structure  Multiculture model with differentiated cells | Damage | *C. albicans* / *C. glabrata:* (Silva *et al.* 2011)  *C. albicans:* (Cavalcanti *et al.* 2015; Mailander-Sanchez *et al.* 2017) |
|  |  | Transcriptomics | *C. albicans:* (Nailis *et al.* 2010; Spiering *et al.* 2010) |
|  |  | Cytokine release | *C. albicans:* (Wagener *et al.* 2012) |
| **RHOE**  **+ Fibroblasts**  (Human primary cells) | 3D structure  Multiculture model with differentiated cells | Bacterial interactions | *C. albicans:* (Bertolini *et al.* 2019; de Carvalho Dias *et al.* 2018; Diaz *et al.* 2012) |
|  |  | Biofilm formation | *C. albicans:* (Sobue *et al.* 2018) |
| **Oral mucosa-on-chip**  **Keratinocytes Gie-No3B11**  (Human cell line)  **+ gingival fibroblasts** (Human cell line) | Collagen embedded fibroblast and Keratinocytes on a porous membrane under flow | Model establishment | (Rahimi *et al.* 2018) |
| **B) Intestinal tract** | | | |
| **Colorectal adenocarnima cells**  **Caco-2**  (Human cell line) | Monoculture | Damage | *C. albicans:* (Wachtler *et al.* 2011a) |
|  |  | Invasion | *C. albicans:* (Goyer *et al.* 2016; Wachtler *et al.* 2011a) |
|  |  | Interactions antagonistic yeasts | *C. albicans*: (Lohith and Anu-Appaiah 2018; Murzyn *et al.* 2010)  *C. glabrata*: (Kunyeit *et al.* 2019) |
|  |  | Cytokine release | *C. albicans:* (Gacser *et al.* 2014; Mao *et al.* 2019; Schirbel *et al.* 2018) |
| **Colorectal adenocarnimo cells**  **HT-29**  (Human cell line) | Monoculture | Adhesion  Invasion  Damage | *C. albicans:* (Deng *et al.* 2015; Garcia *et al.* 2018) |
| **Mucus secreting goblet cells**  **HT-29-MTX**  (Human cell line) | Monoculture | Gene expression | *C. albicans:* (Kavanaugh *et al.* 2014) |
| **Subclone of Caco-2**  **C2BBe1**  (Human cell line) | Monoculture in transwell | Translocation | *C. albicans:* (Allert *et al.* 2018) |
|  |  | Transcriptomics | *C. albicans:* (Bohringer *et al.* 2016) |
| **Caco-2**  (Human cell line)  **+ Raji B cells** (Human cell line) | Co-culture | Adhesion  Invasion | *C. albicans:* (Albac *et al.* 2016) |
| **C2BBe1**  (Human cell line)  **+ HT-29-MTX**  (Human cell line) | Co-culture | Adhesion  Damage | *C. albicans:* (Graf *et al.* 2019) |
| **Intestine-on-Chip**  **C2BBe1**  (Human cell line)  **+ HUVECs**  (Human primary cells)  **+ Monocytes**  (Human primary cells) | Bilayer under flow  (top: C2BBe1  Bottom: HUVECs + Monocytes) | Damage  Translocation | *C. albicans:* (Maurer *et al.* 2019) |
| **C) Vaginal tract** | | | |
| **Human**  **immortalized vaginal mucosal cells**  **VK2/E6E7**  (Human cell line) | Monoculture | Adhesion | *C. albicans:* (Luan *et al.* 2020; Mikamo *et al.* 2018) |
|  |  | Bacterial interactions | *C. albicans:* (Pidwill *et al.* 2018) |
|  |  | Autophagy | *C. albicans:* (Shroff and Reddy 2018) |
|  |  | Cytokine release | *C. albicans:* (Li *et al.* 2017) |
| **Vaginal epidermoid carcinoma cells**  **A431**  (Human cell line) | Monoculture | Damage | *C. albicans:* (Wachtler *et al.* 2011b) |
|  |  | Cytokine release | *C. albicans:* (Richardson *et al.* 2018) |
| **Reconstituted vaginal epithelium**  **RHVE**  (cell line based) | 3D structure  Multiculture model with differentiated cells | Invasion  Damage | *C. albicans:* (Alves *et al.* 2014) |
|  |  | Transcriptomics | *C. glabrata:* (Bernardo *et al.* 2017) |
|  |  | Cytokine release | *C. albicans:* (Schaller *et al.* 2005) |

**References Tab. S3:**

Albac S, Schmitz A, Lopez-Alayon C *et al.* Candida albicans is able to use M cells as a portal of entry across the intestinal barrier in vitro. *Cell Microbiol* 2016;**18**: 195-210.

Allert S, Forster TM, Svensson CM *et al.* Candida albicans-Induced Epithelial Damage Mediates Translocation through Intestinal Barriers. *MBio* 2018;**9**.

Alves CT, Wei XQ, Silva S *et al.* Candida albicans promotes invasion and colonisation of Candida glabrata in a reconstituted human vaginal epithelium. *The Journal of infection* 2014;**69**: 396-407.

Bernardo RT, Cunha DV, Wang C *et al.* The CgHaa1-Regulon Mediates Response and Tolerance to Acetic Acid Stress in the Human Pathogen Candida glabrata. *G3 (Bethesda, Md)* 2017;**7**: 1-18.

Bertolini M, Ranjan A, Thompson A *et al.* Candida albicans induces mucosal bacterial dysbiosis that promotes invasive infection. *PLoS Pathog* 2019;**15**: e1007717.

Bohringer M, Pohlers S, Schulze S *et al.* Candida albicans infection leads to barrier breakdown and a MAPK/NF-kappaB mediated stress response in the intestinal epithelial cell line C2BBe1. *Cellular microbiology* 2016;**18**: 889-904.

Casaroto AR, da Silva RA, Salmeron S *et al.* Candida albicans-Cell Interactions Activate Innate Immune Defense in Human Palate Epithelial Primary Cells via Nitric Oxide (NO) and beta-Defensin 2 (hBD-2). *Cells* 2019;**8**.

Cavalcanti YW, Morse DJ, da Silva WJ *et al.* Virulence and pathogenicity of Candida albicans is enhanced in biofilms containing oral bacteria. *Biofouling* 2015;**31**: 27-38.

de Carvalho Dias K, de Sousa DL, Barbugli PA *et al.* Development and characterization of a 3D oral mucosa model as a tool for host-pathogen interactions. *Journal of microbiological methods* 2018;**152**: 52-60.

Deng K, Chen T, Wu Q *et al.* In vitro and in vivo examination of anticolonization of pathogens by Lactobacillus paracasei FJ861111.1. *Journal of dairy science* 2015;**98**: 6759-66.

Diaz PI, Xie Z, Sobue T *et al.* Synergistic interaction between Candida albicans and commensal oral streptococci in a novel in vitro mucosal model. *Infection and immunity* 2012;**80**: 620-32.

Dongari-Bagtzoglou A, Kashleva H. Candida albicans triggers interleukin-8 secretion by oral epithelial cells. *Microbial pathogenesis* 2003;**34**: 169-77.

Gacser A, Tiszlavicz Z, Nemeth T *et al.* Induction of human defensins by intestinal Caco-2 cells after interactions with opportunistic Candida species. *Microbes and infection* 2014;**16**: 80-5.

Garcia C, Burgain A, Chaillot J *et al.* A phenotypic small-molecule screen identifies halogenated salicylanilides as inhibitors of fungal morphogenesis, biofilm formation and host cell invasion. *Scientific reports* 2018;**8**: 11559.

Goyer M, Loiselet A, Bon F *et al.* Intestinal Cell Tight Junctions Limit Invasion of Candida albicans through Active Penetration and Endocytosis in the Early Stages of the Interaction of the Fungus with the Intestinal Barrier. *PloS one* 2016;**11**: e0149159.

Graf K, Last A, Gratz R *et al.* Keeping Candida commensal - How lactobacilli antagonize pathogenicity of Candida albicans in an in vitro gut model. *Disease models & mechanisms* 2019, DOI 10.1242/dmm.039719.

Ho J, Yang X, Nikou SA *et al.* Candidalysin activates innate epithelial immune responses via epidermal growth factor receptor. *Nature communications* 2019;**10**: 2297.

Kavanaugh NL, Zhang AQ, Nobile CJ *et al.* Mucins suppress virulence traits of Candida albicans. *mBio* 2014;**5**: e01911.

Kumar R, Saraswat D, Tati S *et al.* Novel Aggregation Properties of Candida albicans Secreted Aspartyl Proteinase Sap6 Mediate Virulence in Oral Candidiasis. *Infection and immunity* 2015;**83**: 2614-26.

Kunyeit L, Kurrey NK, Anu-Appaiah KA *et al.* Probiotic Yeasts Inhibit Virulence of Non-albicans Candida Species. *mBio* 2019;**10**.

Li T, Niu X, Zhang X *et al.* Recombinant Human IFNalpha-2b Response Promotes Vaginal Epithelial Cells Defense against Candida albicans. *Frontiers in microbiology* 2017;**8**: 697.

Liu Y, Shetty AC, Schwartz JA *et al.* New signaling pathways govern the host response to C. albicans infection in various niches. *Genome research* 2015;**25**: 679-89.

Liu Y, Solis NV, Heilmann CJ *et al.* Role of retrograde trafficking in stress response, host cell interactions, and virulence of Candida albicans. *Eukaryot Cell* 2014;**13**: 279-87.

Lohith K, Anu-Appaiah KA. Antagonistic effect of Saccharomyces cerevisiae KTP and Issatchenkia occidentalis ApC on hyphal development and adhesion of Candida albicans. *Medical mycology* 2018;**56**: 1023-32.

Luan T, Liu X, Mao P *et al.* The Role of 17β-Estrogen in Candida albicans Adhesion on Human Vaginal Epithelial Cells via FAK Phosphorylation. *Mycopathologia* 2020, DOI 10.1007/s11046-020-00440-1.

Mailander-Sanchez D, Braunsdorf C, Grumaz C *et al.* Antifungal defense of probiotic Lactobacillus rhamnosus GG is mediated by blocking adhesion and nutrient depletion. *PloS one* 2017;**12**: e0184438.

Mao X, Qiu X, Jiao C *et al.* Candida albicans SC5314 inhibits NLRP3/NLRP6 inflammasome expression and dampens human intestinal barrier activity in Caco-2 cell monolayer model. *Cytokine* 2019;**126**: 154882.

Maurer M, Gresnigt MS, Last A *et al.* A three-dimensional immunocompetent intestine-on-chip model as in vitro platform for functional and microbial interaction studies. *Biomaterials* 2019;**220**: 119396.

McCall AD, Kumar R, Edgerton M. Candida albicans Sfl1/Sfl2 regulatory network drives the formation of pathogenic microcolonies. *PLoS pathogens* 2018;**14**: e1007316-e.

Mikamo H, Yamagishi Y, Sugiyama H *et al.* High glucose-mediated overexpression of ICAM-1 in human vaginal epithelial cells increases adhesion of Candida albicans. *Journal of obstetrics and gynaecology : the journal of the Institute of Obstetrics and Gynaecology* 2018;**38**: 226-30.

Morse DJ, Wilson MJ, Wei X *et al.* Denture-associated biofilm infection in three-dimensional oral mucosal tissue models. *Journal of medical microbiology* 2018;**67**: 364-75.

Murzyn A, Krasowska A, Augustyniak D *et al.* The effect of Saccharomyces boulardii on Candida albicans-infected human intestinal cell lines Caco-2 and Intestin 407. *FEMS microbiology letters* 2010;**310**: 17-23.

Nailis H, Kucharikova S, Ricicova M *et al.* Real-time PCR expression profiling of genes encoding potential virulence factors in Candida albicans biofilms: identification of model-dependent and -independent gene expression. *BMC microbiology* 2010;**10**: 114.

Offenbacher S, Barros SP, Bencharit S *et al.* Differential Mucosal Gene Expression Patterns in Candida-Associated, Chronic Oral Denture Stomatitis. *Journal of prosthodontics : official journal of the American College of Prosthodontists* 2019;**28**: 202-8.

Pidwill GR, Rego S, Jenkinson HF *et al.* Coassociation between Group B Streptococcus and Candida albicans Promotes Interactions with Vaginal Epithelium. *Infection and immunity* 2018;**86**.

Puri S, Kumar R, Rojas IG *et al.* Iron Chelator Deferasirox Reduces Candida albicans Invasion of Oral Epithelial Cells and Infection Levels in Murine Oropharyngeal Candidiasis. *Antimicrobial agents and chemotherapy* 2019;**63**.

Rahimi C, Rahimi B, Padova D *et al.* Oral mucosa-on-a-chip to assess layer-specific responses to bacteria and dental materials. *Biomicrofluidics* 2018;**12**: 054106.

Richardson JP, Willems HME, Moyes DL *et al.* Candidalysin Drives Epithelial Signaling, Neutrophil Recruitment, and Immunopathology at the Vaginal Mucosa. *Infection and immunity* 2018;**86**.

Schaller M, Korting HC, Borelli C *et al.* Candida albicans-secreted aspartic proteinases modify the epithelial cytokine response in an in vitro model of vaginal candidiasis. *Infection and immunity* 2005;**73**: 2758-65.

Schaller M, Schafer W, Korting HC *et al.* Differential expression of secreted aspartyl proteinases in a model of human oral candidosis and in patient samples from the oral cavity. *Molecular microbiology* 1998;**29**: 605-15.

Schirbel A, Shouval DS, Hebecker B *et al.* Intestinal epithelial cells and T cells differentially recognize and respond to Candida albicans yeast and hypha. *European journal of immunology* 2018;**48**: 1826-37.

Shroff A, Reddy KVR. Autophagy gene ATG5 knockdown upregulates apoptotic cell death during Candida albicans infection in human vaginal epithelial cells. *American journal of reproductive immunology (New York, NY : 1989)* 2018;**80**: e13056.

Silva S, Henriques M, Hayes A *et al.* Candida glabrata and Candida albicans co-infection of an in vitro oral epithelium. *Journal of oral pathology & medicine : official publication of the International Association of Oral Pathologists and the American Academy of Oral Pathology* 2011;**40**: 421-7.

Sobue T, Bertolini M, Thompson A *et al.* Chemotherapy-induced oral mucositis and associated infections in a novel organotypic model. *Molecular oral microbiology* 2018;**33**: 212-23.

Solis NV, Park YN, Swidergall M *et al.* Candida albicans White-Opaque Switching Influences Virulence but Not Mating during Oropharyngeal Candidiasis. *Infection and immunity* 2018;**86**.

Solis NV, Swidergall M, Bruno VM *et al.* The Aryl Hydrocarbon Receptor Governs Epithelial Cell Invasion during Oropharyngeal Candidiasis. *mBio* 2017;**8**.

Spiering MJ, Moran GP, Chauvel M *et al.* Comparative transcript profiling of Candida albicans and Candida dubliniensis identifies SFL2, a C. albicans gene required for virulence in a reconstituted epithelial infection model. *Eukaryotic cell* 2010;**9**: 251-65.

Swidergall M, Solis NV, Lionakis MS *et al.* EphA2 is an epithelial cell pattern recognition receptor for fungal β-glucans. *Nature microbiology* 2018;**3**: 53-61.

Verma AH, Richardson JP, Zhou C *et al.* Oral epithelial cells orchestrate innate type 17 responses to Candida albicans through the virulence factor candidalysin. *Science immunology* 2017;**2**.

Verma AH, Zafar H, Ponde NO *et al.* IL-36 and IL-1/IL-17 Drive Immunity to Oral Candidiasis via Parallel Mechanisms. *J Immunol* 2018;**201**: 627-34.

Villar CC, Kashleva H, Nobile CJ *et al.* Mucosal tissue invasion by Candida albicans is associated with E-cadherin degradation, mediated by transcription factor Rim101p and protease Sap5p. *Infection and immunity* 2007;**75**: 2126-35.

Wachtler B, Wilson D, Haedicke K *et al.* From attachment to damage: defined genes of Candida albicans mediate adhesion, invasion and damage during interaction with oral epithelial cells. *PloS one* 2011a;**6**: e17046.

Wachtler B, Wilson D, Hube B. Candida albicans adhesion to and invasion and damage of vaginal epithelial cells: stage-specific inhibition by clotrimazole and bifonazole. *Antimicrobial agents and chemotherapy* 2011b;**55**: 4436-9.

Wagener J, Mailander-Sanchez D, Schaller M. Immune responses to Candida albicans in models of in vitro reconstituted human oral epithelium. *Methods Mol Biol* 2012;**845**: 333-44.

Wilson D, Mayer FL, Miramon P *et al.* Distinct roles of Candida albicans-specific genes in host-pathogen interactions. *Eukaryot Cell* 2014;**13**: 977-89.

Wollert T, Rollenhagen C, Langford GM *et al.* Human oral keratinocytes: a model system to analyze host-pathogen interactions. *Methods in molecular biology (Clifton, NJ)* 2012;**845**: 289-302.

Zhu W, Phan QT, Boontheung P *et al.* EGFR and HER2 receptor kinase signaling mediate epithelial cell invasion by Candida albicans during oropharyngeal infection. *Proceedings of the National Academy of Sciences of the United States of America* 2012;**109**: 14194-9.

**Table S4: Overview about the different endothelial and blood-brain barrier *in vitro* models, their structure, possible read outs and applications for the different fungi**

| **Model** | **Structure** | **Readout** | **Reference** |
| --- | --- | --- | --- |
| **Human umbilical vein endothelial cells HUVECs**  (Human primary cells) | Monoculture | Adhesion | *C. albicans:* (Citiulo *et al.* 2012; Lopez *et al.* 2014; Phan *et al.* 2013)  *C. neoformans*: (Ibrahim *et al.* 1995) |
|  |  | Invasion | *A. fumigatus:* (Liu et al. 2016)  *C. albicans:* (Phan *et al.* 2013; Phan *et al.* 2005; Phan *et al.* 2007) |
|  |  | Damage | *A. fumigatus:* (Ejzykowicz et al. 2010)  *C. albicans:* (Rotrosen et al. 1985; Sanchez et al. 2004) |
|  |  | Proteomics | *A. fumigatus:* (Neves *et al.* 2016; Zhang *et al.* 2017b)  *C. neoformans*: (Wang *et al.* 2011) |
|  |  | Transcriptomics | *C. albicans:* (Barker et al. 2008)  *C. neoformans*: (Coenjaerts *et al.* 2006) |
|  |  | Cytokine release | *A.fumigatus:* (Kamai *et al.* 2009; Neves *et al.* 2017)  *C. albicans*: (Orozco *et al.* 2000) |
|  | Monoculture under flow | Adhesion | *C. albicans:* (Wilson and Hube 2010) |
| **HUVECs**  (Human primary cells)  +  **Neutrophils**  (Human primary cells) | Co-culture | Damage | *C. albicans:* (Edwards et al. 1987) |
| **Human brain microvascular endothelial cells**  **HMBEC**  **HCMEC/D3**  (Human cell line) | Monoculture on Transwell | Adhesion  Invasion | *C. albicans*: (Liu *et al.* 2011) |
|  |  | TEER  Permeability assay | *A. fumigatus*: (Patel *et al.* 2018)  *C. neoformans:* (Stie and Fox 2012) |
|  |  | Traversal | *C. neoformans:* (Aaron *et al.* 2018; Chang *et al.* 2004; Huang *et al.* 2011; Kim *et al.* 2012; Na Pombejra *et al.* 2018; Na Pombejra *et al.* 2017; Vu *et al.* 2013; Vu *et al.* 2009; Zhu *et al.* 2017) |
|  |  | TEM | *C. neoformans*: (Chen *et al.* 2003) |
|  |  | Transcriptomics | *C. neoformans*: (Lahiri *et al.* 2019) |
| **HMBEC**  **HCMEC/D3**  (Human cell line)  **+ THP-1**  (Human cell line)  **or Monocytes**  (Human primary cells) | Co-culture on transwell | Model development | *C. neoformans*: (Santiago-Tirado *et al.* 2019) |
|  |  | Traversal | *C. neoformans*: (He *et al.* 2016; Santiago-Tirado *et al.* 2017; Sorrell *et al.* 2016) |
| **BBB -on-Chip**  **HUVECs**  (Human primary cells) | Monoculture under flow | Model establishment | (Yeon *et al.* 2012) |
| **BBB -on-Chip**  **HCMEC/D3**  (Human cell line) | Monoculture under flow | Model establishment | (Griep *et al.* 2013) |
| **BBB -on-Chip**  **HCMEC/D3**  **+ astrocytes**  (Human primary cells)  **+ pericytes**  (Human primary cells)  **+ fibrobasts**  (Human primary cells) | Multiculture under flow | Model establishment | (Lee *et al.* 2020) |

**References Tab. S4:**

Aaron PA, Jamklang M, Uhrig JP *et al.* The blood-brain barrier internalises Cryptococcus neoformans via the EphA2-tyrosine kinase receptor. *Cellular microbiology* 2018;**20**.

Barker KS, Park H, Phan QT *et al.* Transcriptome profile of the vascular endothelial cell response to Candida albicans. *The Journal of infectious diseases* 2008;**198**: 193-202.

Chang YC, Stins MF, McCaffery MJ *et al.* Cryptococcal yeast cells invade the central nervous system via transcellular penetration of the blood-brain barrier. *Infection and immunity* 2004;**72**: 4985-95.

Chen SHM, Stins MF, Huang SH *et al.* Cryptococcus neoformans induces alterations in the cytoskeleton of human brain microvascular endothelial cells. *Journal of medical microbiology* 2003;**52**: 961-70.

Citiulo F, Jacobsen ID, Miramon P *et al.* Candida albicans scavenges host zinc via Pra1 during endothelial invasion. *PLoS Pathog* 2012;**8**: e1002777.

Coenjaerts FE, Hoepelman AI, Scharringa J *et al.* The Skn7 response regulator of Cryptococcus neoformans is involved in oxidative stress signalling and augments intracellular survival in endothelium. *FEMS yeast research* 2006;**6**: 652-61.

Edwards JE, Jr., Rotrosen D, Fontaine JW *et al.* Neutrophil-mediated protection of cultured human vascular endothelial cells from damage by growing Candida albicans hyphae. *Blood* 1987;**69**: 1450-7.

Ejzykowicz DE, Solis NV, Gravelat FN *et al.* Role of Aspergillus fumigatus DvrA in host cell interactions and virulence. *Eukaryot Cell* 2010;**9**: 1432-40.

Griep LM, Wolbers F, de Wagenaar B *et al.* BBB on chip: microfluidic platform to mechanically and biochemically modulate blood-brain barrier function. *Biomed Microdevices* 2013;**15**: 145-50.

He X, Shi X, Puthiyakunnon S *et al.* CD44-mediated monocyte transmigration across Cryptococcus neoformans-infected brain microvascular endothelial cells is enhanced by HIV-1 gp41-I90 ectodomain. *J Biomed Sci* 2016;**23**: 28.

Huang SH, Long M, Wu CH *et al.* Invasion of Cryptococcus neoformans into human brain microvascular endothelial cells is mediated through the lipid rafts-endocytic pathway via the dual specificity tyrosine phosphorylation-regulated kinase 3 (DYRK3). *J Biol Chem* 2011;**286**: 34761-9.

Ibrahim AS, Filler SG, Alcouloumre MS *et al.* Adherence to and damage of endothelial cells by Cryptococcus neoformans in vitro: role of the capsule. *Infection and immunity* 1995;**63**: 4368-74.

Kamai Y, Lossinsky AS, Liu H *et al.* Polarized response of endothelial cells to invasion by Aspergillus fumigatus. *Cellular microbiology* 2009;**11**: 170-82.

Kim JC, Crary B, Chang YC *et al.* Cryptococcus neoformans activates RhoGTPase proteins followed by protein kinase C, focal adhesion kinase, and ezrin to promote traversal across the blood-brain barrier. *J Biol Chem* 2012;**287**: 36147-57.

Lahiri S, Banerjee A, Bhutda S *et al.* In vitro expression of vital virulent genes of clinical and environmental isolates of Cryptococcus neoformans/gattii in endothelial cells of human blood-brain barrier. *J Mycol Med* 2019;**29**: 239-44.

Lee S, Chung M, Lee SR *et al.* 3D brain angiogenesis model to reconstitute functional human blood-brain barrier in vitro. *Biotechnol Bioeng* 2020;**117**: 748-62.

Liu H, Lee MJ, Solis NV *et al.* Aspergillus fumigatus CalA binds to integrin alpha5beta1 and mediates host cell invasion. *Nature microbiology* 2016;**2**: 16211.

Liu Y, Mittal R, Solis NV *et al.* Mechanisms of Candida albicans trafficking to the brain. *PLoS pathogens* 2011;**7**: e1002305.

Lopez CM, Wallich R, Riesbeck K *et al.* Candida albicans uses the surface protein Gpm1 to attach to human endothelial cells and to keratinocytes via the adhesive protein vitronectin. *PloS one* 2014;**9**: e90796.

Na Pombejra S, Jamklang M, Uhrig JP *et al.* The structure-function analysis of the Mpr1 metalloprotease determinants of activity during migration of fungal cells across the blood-brain barrier. *PloS one* 2018;**13**: e0203020.

Na Pombejra S, Salemi M, Phinney BS *et al.* The Metalloprotease, Mpr1, Engages AnnexinA2 to Promote the Transcytosis of Fungal Cells across the Blood-Brain Barrier. *Frontiers in cellular and infection microbiology* 2017;**7**: 296.

Neves GW, Curty N, Kubitschek-Barreira PH *et al.* Dataset of differentially regulated proteins in HUVECs challenged with wild type and UGM1 mutant Aspergillus fumigatus strains. *Data in brief* 2016;**9**: 24-31.

Neves GW, Curty NA, Kubitschek-Barreira PH *et al.* Modifications to the composition of the hyphal outer layer of Aspergillus fumigatus modulates HUVEC proteins related to inflammatory and stress responses. *Journal of proteomics* 2017;**151**: 83-96.

Orozco AS, Zhou X, Filler SG. Mechanisms of the proinflammatory response of endothelial cells to Candida albicans infection. *Infection and immunity* 2000;**68**: 1134-41.

Patel R, Hossain MA, German N *et al.* Gliotoxin penetrates and impairs the integrity of the human blood-brain barrier in vitro. *Mycotoxin research* 2018;**34**: 257-68.

Phan QT, Eng DK, Mostowy S *et al.* Role of endothelial cell septin 7 in the endocytosis of Candida albicans. *mBio* 2013;**4**: e00542-13.

Phan QT, Fratti RA, Prasadarao NV *et al.* N-cadherin mediates endocytosis of Candida albicans by endothelial cells. *J Biol Chem* 2005;**280**: 10455-61.

Phan QT, Myers CL, Fu Y *et al.* Als3 is a Candida albicans invasin that binds to cadherins and induces endocytosis by host cells. *PLoS Biol* 2007;**5**: e64.

Rotrosen D, Edwards JE, Jr., Gibson TR *et al.* Adherence of Candida to cultured vascular endothelial cells: mechanisms of attachment and endothelial cell penetration. *The Journal of infectious diseases* 1985;**152**: 1264-74.

Sanchez AA, Johnston DA, Myers C *et al.* Relationship between Candida albicans virulence during experimental hematogenously disseminated infection and endothelial cell damage in vitro. *Infection and immunity* 2004;**72**: 598-601.

Santiago-Tirado FH, Klein RS, Doering TL. An In Vitro Brain Endothelial Model for Studies of Cryptococcal Transmigration into the Central Nervous System. *Current protocols in microbiology* 2019;**53**: e78.

Santiago-Tirado FH, Onken MD, Cooper JA *et al.* Trojan Horse Transit Contributes to Blood-Brain Barrier Crossing of a Eukaryotic Pathogen. *mBio* 2017;**8**.

Sorrell TC, Juillard PG, Djordjevic JT *et al.* Cryptococcal transmigration across a model brain blood-barrier: evidence of the Trojan horse mechanism and differences between Cryptococcus neoformans var. grubii strain H99 and Cryptococcus gattii strain R265. *Microbes and infection* 2016;**18**: 57-67.

Stie J, Fox D. Blood-brain barrier invasion by Cryptococcus neoformans is enhanced by functional interactions with plasmin. *Microbiology (Reading, England)* 2012;**158**: 240-58.

Vu K, Eigenheer RA, Phinney BS *et al.* Cryptococcus neoformans promotes its transmigration into the central nervous system by inducing molecular and cellular changes in brain endothelial cells. *Infection and immunity* 2013;**81**: 3139-47.

Vu K, Weksler B, Romero I *et al.* Immortalized human brain endothelial cell line HCMEC/D3 as a model of the blood-brain barrier facilitates in vitro studies of central nervous system infection by Cryptococcus neoformans. *Eukaryotic cell* 2009;**8**: 1803-7.

Wang XJ, Zhu YJ, Cui JG *et al.* Proteomic analysis of human umbilical vein endothelial cells incubated with Cryptococcus neoformans var. neoformans. *Mycoses* 2011;**54**: e336-43.

Wilson D, Hube B. Hgc1 mediates dynamic Candida albicans-endothelium adhesion events during circulation. *Eukaryot Cell* 2010;**9**: 278-87.

Yeon JH, Na D, Choi K *et al.* Reliable permeability assay system in a microfluidic device mimicking cerebral vasculatures. *Biomed Microdevices* 2012;**14**: 1141-8.

Zhang PP, Xin XF, Xu XY *et al.* Toll-like receptor 2 and dectin-1 function as promising biomarker for Aspergillus fumigatus infection. *Experimental and therapeutic medicine* 2017;**14**: 3836-40.

Zhu L, Maruvada R, Sapirstein A *et al.* Cysteinyl leukotrienes as novel host factors facilitating Cryptococcus neoformans penetration into the brain. *Cellular microbiology* 2017;**19**.
